# Synthesis and Antifungal Activity of Benzamidine Derivatives Carrying 1,2,3-Triazole Moieties

**DOI:** 10.3390/molecules19055674

**Published:** 2014-05-02

**Authors:** Guangyou Chen, Yiwan Zhou, Chonglin Cai, Jia Lu, Xing Zhang

**Affiliations:** Research & Development Center of Biorational Pesticide, Key Laboratory of Plant Protection Resources and Pest Management of Ministry of Education, Northwest A&F University, Yangling 712100, China

**Keywords:** amidine, 1,2,3-triazole, antifungal activity, synthesis

## Abstract

Eighteen novel benzamidine derivatives containing 1,2,3-triazole moieties were synthesized. The *in vitro* and *in vivo* fungicidal acitivities of the title compounds and the arylamidine intermediates against *Colletotrichum lagenarium* and *Botrytis cinerea* were tested. The synthesized benzamidines exhibited weak antifungal activities *in vitro* against the tested fungi, but some of the compounds showed excellent activities *in vivo* to the same strains. Among the compounds tested, **9b** showed 79% efficacy *in vivo* against *C. lagenarium* at a concentration of 200 μg/mL, and the efficacy of compound **16d** (90%) toward the same strain was even superior than that of the commercial fungicide carbendazim (85%).

## 1. Introduction

The majority of agricultural phytopathogens, especially *Botrytis cinerea*, the causal agent of the common disease gray mold on vegetable and fruit crops, have developed resistance to the most commonly used fungicides all over the world [1]. Application of synthetic fungicides is still the most efficient tool to control crop diseases. This situation urged us to find new compounds having agricultural antifungal activities. Amidine derivatives exhibit several significant bioactivities, such as antitumor [2], trypanocidal [3,4,5], antiprotozoan [6,7], anti-HIV [8], diuretic, anti-inflammatory, analgesic [9], antivirus, fungicidal and bactericidal [10,11,12,13] activities. Amidines can also be used in the synthesis of metallo-organic compounds [14]. Aromatic diamidines has been explored as agents against a widespread range of microorganisms [15]. In our previous works, aromatic diamidines were found to have excellent antifungal activities in agriculture, especially for the treatment of gray mold on tomatoes [16,17]. It was reported that good bioacitivities could be obtained by linking the arylamidino group with heteorocycle moieties [18,19,20]. 

1,2,3-Triazoles are very important heterocycles showing excellent bioactivities in pharmaceutical and agrochemical applications. Compounds containing 1,2,3-triazoles have been reported for their antifungal [21,22,23], antibacterial [24,25,26,27], anti-HIV [28], insecticidal [29], herbicidal [30], antiviral [31], antitumor [32], tuberculosis inhibitory [33], antiprotozoal [34], antimalarial [35], anticancer [36], and larvicidal [37] activities. The fungicidal activity of the commercial fungicide fluconazole can be improved by introducing a 1,2,3-triazole moiety into the molecule [38,39]. The presence of the 1,2,3-triazole moiety in oxazolidinone compounds can increase the protein binding ability and subsequently improve the antibacterial activities [40]. The compounds formed by linking a 1,2,3-triazole ring and an indole nucleus together possessed much higher antimicrobial activity compared with the precursors [41]. Another advantage of 1,2,3-triazoles is that they can be prepared conveniently by Huisgen’s cycloaddition reaction between azides and terminal alkynes without complicated purification. 

In an attempt to find more potent antifungal molecules, a series of new compounds were designed by linking aromatic amidines and 1,2,3-triazoles. In addition, their *in vitro* and *in vivo* biological activity toward the strains *C. lagenarium* and *B. cinerea* were studied in this paper.

## 2. Results and Discussion

### 2.1. Chemistry

**Scheme 1:**
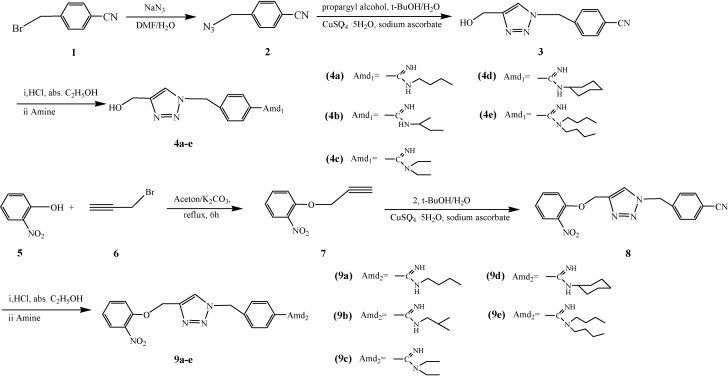
Synthesis of compounds **4** and **9**.

Target molecules **4a**–**e** were synthesized according to Scheme 1. 4-(Azidomethyl)benzonitrile (**2**) was obtained by adding aqueous sodium azide to 4-cyanobenzyl bromide in DMF [42], the reaction took place quantitatively and the product can be separated by simple extraction. The potentially explosive benzyl azide **2** should be handled carefully and the precipitation should be avoided by first adding toluene to the reaction mixture in the end of the reaction. After being dried, **2** can be used as a solution without evaporation of the solvent. Then compound **2** was reacted with propargyl alcohol under standard “click chemistry” [43] conditions using a copper sulfate/sodium ascorbate system as the catalyst. The copper sulfate and sodium ascorbate were dissolved in water before being added to the reaction mixture to ensure a good dispersion of the resultant cuprous salt. The temperature is crucial in this step. Little **2** had been converted and the mixture stayed clear when the reaction mixture being stirred for 48 h at 40 °C. However, plenty of crystals were formed in the flask after being stirred for 6 h at 55 °C when TLC indicated that no **2** remained. The target compound was obtained in high yield by recrystallization. 4-((4-(Hydroxymethyl)-1*H*-1,2,3-triazol-1-yl) methyl)benzonitrile (**3**) was converted into **4** by a Pinner reaction [44]. Anhydrous conditions were important in the synthesis of the Pinner salt. Compound **3** was dried under 110 °C before use, and absolute ethanol was adopted as both solvent and reactant. The moisture-sensitive Pinner salt was handled quickly when being filtered and recrystallized in order to reduce byproducts. The Pinner salt was dissolved in absolute ethanol and kept under anhydrous conditions before use to avoid the absorption of moisture. Compounds **4a**–**e** were obtained by the ammonization of the Pinner salt with corresponding amines.

Compounds **9a**–**e** were obtained according to the procedure described in Scheme 1. 4-((4-((2-Nitrophenoxy)methyl)-1*H*-1,2,3-triazol-1-yl)methyl)-benzonitrile (**8**) was obtained by reacting 1-nitro-2-(prop-2-ynyloxy) benzene (**7**) with **2** under “click chemistry” conditions. Compound **8** was converted into the corresponding amidines **9** following the same procedure used for **4**.

**Scheme 2:**
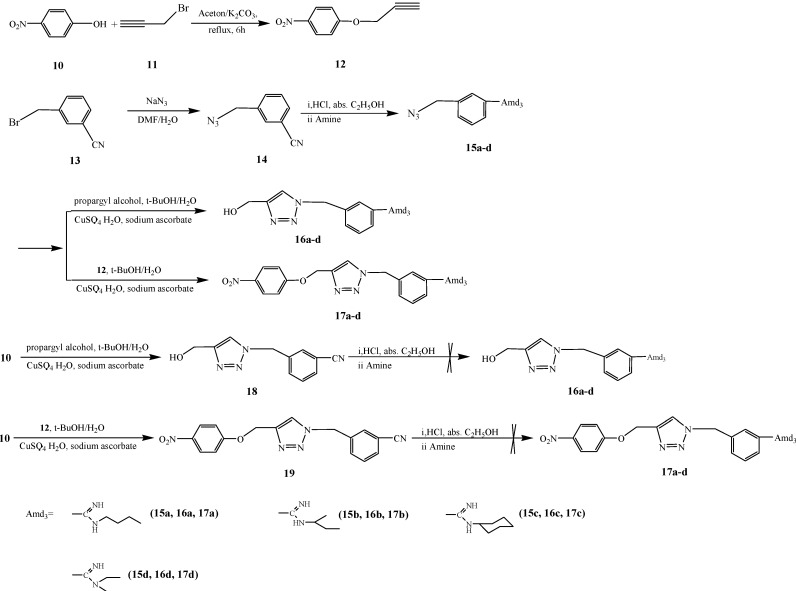
Synthesis of compounds **16** and **17**.

When we applied the method used for the preparation of **4** and **9** in synthesizing **16** and **17**, the target products could not be obtained. Compounds **18** and **19** were obtained in high yields by reacting **10** with propargyl alcohol or **12**, respectively. However, when treated with hydrogen chloride in anhydrous ethanol, the cyano group on **18** or **19** could not be converted into the corresponding Pinner salt. We surmised that the unreactiveness of the cyano group on the 3-position of the benzene ring may be attributed to the electron donating aminomethyl group at the *meta* position [45], which decreased the electron density at the position the cyano group is linked to, and made it very difficult for the HCl-catalyzed Pinner reaction [46] to occur. The synthesis strategy was therefore adjusted as shown in Scheme 2. The 3-(azidomethyl)benzonitrile was inverted into the corresponding amidines by a Pinner reaction [42] before the 3-(azidomethyl)-N-benzamidines **15a**–**e** were connected with 1-nitro-4-(prop-2-ynyloxy)benzene (**12**) or propargyl alcohol using the “click chemistry” method to obtain the target compounds.

The chemical structures of the target compounds (Table 1) were confirmed by their spectral data (^1^H-NMR, ^13^C-NMR, ESI-MS, and elementary analysis). The ^1^H-NMR spectra of compounds **4**, **9**, **16** and **17** showed a single NH signal from the triazole group at 7.95–8.25 ppm [47,48] and the signal of CH (or CH_2_) attached to the amidines at 2.97–3.89 ppm [49]. The ^13^C-NMR spectra of the compounds **4**, **9**, **15**, **16** and **17** showed the signal of C=N in the amidino group at 162–166 ppm [50]. Based on the abovementioned data, it can be concluded that the structures of the amidines were identified correctly.

**Table 1:** Inhibition activity (%) of the amidines on phytopathogenic fungi ^a^.

Compounds	Inhibition rate (%)			
	*in vitro* (200 μg/mL)		*in vivo* (300 μg/mL)	
	*C. lagenarium*	*B. cinerea*	*C. lagenarium*	*B. cinerea*
**4a**	26	23	49	19
**4b**	19	14	30	60
**4c**	17	25	60	23
**4d**	8	0	0	16
**4e**	10	12	48	56
**9a**	32	22	57	24
**9b**	31	30	79	49
**9c**	61	17	4	13
**9d**	29	44	17	35
**9e**	35	14	45	14
**15a**	12	16	31	43
**15b**	25	19	61	68
**15c**	12	24	0	48
**15d**	10	13	0	46
**16a**	25	15	19	11
**16b**	16	8	56	32
**16c**	10	5	52	47
**16d**	26	13	90	7
**17a**	65	36	52	74
**17b**	21	19	27	0
**17c**	32	12	45	45
**17d**	10	17	53	31
**Carbendazim**	100	100	85	61

^a^ The data are the average of three duplicated results.

### 2.2. Antifungal Activity Bioassay

The *in vitro* and *in vivo* fungicidal activities of the amidines **4a**–**e**, **9a**–**e**, **16a**–**d**, **17a**–**d** and **15a**–**d** are listed in Table 1. Some of the compounds showed valuable *in vitro* inhibitory activity against *C. lagenarium* and *B. cinerea* at a concentration of 200 μg/mL. The test results showed that some of the title compounds and the intermediates 3-(azidomethyl)-N-benzamidine **15a**–**d** possessed fairly potent antifungal activities *in vivo* to both *C. lagenarium* and *B. cinerea* at the concentration of 300 μg/mL. The antifungal activity of those compounds was compared with that of the the commercial fungicide carbendazim at the same concentration. Compounds **9c** and **17a** exhibited a significant *in vitro* inhibition effect against *C. lagenarium*, but the inhibition activities (control efficacy of 61% and 65%) were less than those of the fungicide carbendazim (100%). The analysis above indicated that the variation of the substituent groups on the 1,2,3-triazole ring had a great effect on the antifungal activity against *C. lagenarium*. The activities of most compounds in the **9** and **17** series having nitrophenoxyl groups on the 1,2,3-triazole ring were higher than those of the **4** and **16** series with hydroxymethyl ones. For example, the activities of compounds **4c** and **4d** were 17% and 9%, while the activities of **9c** and **9d** were increased to 61% and 29%. The cyclization of azido groups with propargyl alcohol had no obvious effect on the antifungal activities. However, when propargyl alcohol was replaced with 1-nitro-4-(prop-2-ynyloxy)benzene, the activities were increased significantly. The substituents on the amidino group and the position of the amidino group on the phenyl ring also had significant effects on the antifungal activities, but no rules could be extracted from the activity data. The *in vitro* activity of the compounds to *B. cinerea* obeyed the same rule, however, it was not as obvious as the one drawn from the *C. lagenarium* data. Some of the compounds exhibited significant activities *in vivo* toward *C. lagenarium*, in which the compounds **4c**, **9a**, **9b**, **16d** and **15b** had control efficacy rates of 60%, 57%, 79% and 61%, respectively. It was noteworthy that compound **16d** was found to be even more effective than the fungicide carbendazim (control efficacy of 85%). For *B. cinerea*, many compounds showed excellent *in vivo* antifungal activities. Compounds **4b**, **4e**, and **17a** and **15b**, whose efficacy rates were 60%, 56%, 75%, and 68%, respectively, were found to be much more effective as compared to the fungicide carbendazim SC (54%) against *B. cinerea*. The different substituents at the 4-position of the triazole ring and amidino group had no obvious relationship with the fungicidal activity against *B. cinerea*. Most of the compounds exhibited more effective activities *in vivo*, especially for *B. cinerea*, than ones *in vitro* when compared with the commercial fungicide. The aryl amidine moieties played a very crucial role in the antifungal activities.

## 3. Experimental

### 3.1. General Information

Melting points were measured on an X-4 melting point apparatus (Beijing Tech. Instrument Co., Beijing, China), and were not corrected. Mass spectra were obtained on a Thermo-Finnigan LCQ-Advantage (ESI) instrument (Thermo Electron Corporation, Waltham, MA, USA); ^1^H-NMR and ^13^C-NMR spectra were obtained on Bruker 400 MHz or 500 MHz spectrometers (Bruker Corporation, Billerica, MA, USA) in CDCl_3_, CDOD_3_, or (CD_3_)_2_SO solution; elemental analysis was performed on an Elemental Vario-III CHN analyzer (Elementar Analysensysteme GmbH, Hanau, Germany). Reagents and solvents were all chemically or analytically pure without further purification if not mentioned. Anhydrous solvents were purified by standard techniques before use. Anhydrous HCl (gas) was prepared by the addition of concentrated hydrochloric acid dropwise to concentrated sulfuric acid and dried by the latter. Reaction progress was monitored by TLC on silica gel F254 plates prepared in the laboratory, and was visualized under UV_254_ and iodine.

### 3.2. Synthesis of 4-(Azidomethyl)benzonitrile (**2**) [42]

4-Cyanobenzyl bromide (**1**, 45.10 g, 230 mmol) was dissolved in DMF (200 mL) in a flask equipped with a thermometer, and a solution of NaN_3_ (19.80 g, 300 mmol) in distilled water (100 mL) was added to it in batches under ice bath cooling with stirring, keeping the reaction mixture under 15 °C. The reaction mixture was stirred for 2 more h at ambient temperature after the addition of the sodium azide until TLC indicated no **1** remained. The mixture was transferred to a conical flask and diluted with toluene (200 mL) and distilled water (600 mL). The mixture was separated in a separating funnel and the aqueous phase was extracted with toluene (2 × 70 mL). The organic extracts were combined and washed with brine (70 mL) and water (2 × 70 mL) and dried by MgSO_4_ overnight. A solution of **2** in toluene (310 mL) was obtained after filtration. After the solvent was evaporated under reduced pressure, **2** was obtained as light yellow oil (35.69 g, 98.2%), ^1^H-NMR (500 MHz, CDCl_3_) *δ*: 7.61 (d, *J* = 8.2 Hz, 2H, benzene-H), 7.41 (d, *J* = 7.4 Hz, 2H, benzene-H), 4.42 (s, 2H, CH_2_). ^13^C-NMR (125 MHz, CDCl_3_) *δ*: 140.97, 132.82, 132.55, 132.55, 129.07, 128.56, 128.56, 128.27, 125.35, 118.55, 111.92, 77.80, 77.55, 77.29, 53.90, 53.90, 21.37.

### 3.3. Synthesis of 4-((4-(Hydroxymethyl)-1H-1,2,3-triazol-1-yl)methyl) Benzonitrile (**3**)

Compound **2** (35.1 g) was dissolved in a mixture of *t*-BuOH (300 mL) and distilled water (100 mL), then propargyl alcohol (12.3 g, 230 mmol) was added. The reaction mixture was stirred in a water bath at 60 °C for 48 h after the addition of sodium ascorbate (306 mg, in 3 mL of water) and cupric sulfate (CuSO_4_·5H_2_O) (105 mg, in 2 mL of water). The clear reaction mixture turned light yellow. Compound **3** (46.65 g, 128 mmol, 81%) was obtained as white needle-like crystals after filtration and dried under 110 °C for 7 h when most of the solvent was evaporated under vacuum; m.p. 136–137 °C. ^1^H-NMR (400 MHz, DMSO) *δ*: 8.09 (s, 1H, triazole-H), 7.86 (d, *J* = 8.3 Hz, 2H, benzene-H), 7.46 (d, *J* = 8.3 Hz, 2H, benzene-H), 5.70 (s, 2H, CH_2_), 5.22 (t, *J* = 5.6 Hz, 1H, OH), 4.53 (d, *J* = 5.4 Hz, 2H, CH_2_). ^13^C-NMR (100 MHz, DMSO) *δ*: 148.97, 142.19, 133.19, 129.10, 123.73, 119.04, 111.34, 55.49, 52.53.

### 3.4. General Procedure for the Synthesis of 4-((4-(Hydroxymethyl)-1H-1,2,3-triazol-1-yl) Methyl)-benzamidines **4a**–**e**

A suspension of **3** (3.0 g, 14.0 mmol) in absolute ethanol (50 mL) was cooled to below 5 °C and saturated with dry HCl gas. Then the mixture was sealed and stirred at ambient temperature for 48 h. White crystalline imino ester was obtained after evaporation of the solvent under anhydrous conditions and washing with dry ether (3 × 15 mL). The resulting imino ester was dissolved in absolute methanol (20 mL). An exothermic reaction took place and the mixture became clear when it was treated with the corresponding amines (60 mmol). Keeping the reaction mixture stirring overnight in sealed flasks resulted in the formation of the corresponding amidines. After evaporation of the solvent in anhydrous conditions, the residue was dissolved in a mixture of ethanol (5 mL) and 2 N aqueous NaOH (5 mL). The target product was precipitated by the addition of ether (40 mL) to the organic phase and then purified by column chromatography on silica gel (CH_2_Cl_2_/MeOH = 6:1). Compounds **4a**–**d** were prepared by the same method. 

*N-Butyl-4-((4-(hydroxymethyl)-1H-1,2,3-triazol-1-yl)methyl)benzamidine* (**4a**): Yield, 42.1%; white crystals; m.p. 111–112 °C. ^1^H-NMR (400 MHz, MeOD) *δ*: 8.05 (d, *J* = 12.1 Hz, 1H, triazole-H), 7.75 (d, *J* = 8.2 Hz, 2H, benzene-H), 7.53 (d, *J* = 8.2 Hz, 2H, benzene-H), 5.75 (s, 2H, CH_2_), 5.69 (d, *J* = 6.0 Hz, 2H, CH_2_), 3.42 (t, *J* = 7.3 Hz, 2H, N-CH_2_), 1.83–1.73 (m, 2H, CH_2_), 1.05 (t, *J* = 7.4 Hz, 3H, CH_3_). ^13^C-NMR (100 MHz, MeOD) *δ*: 166.56, 141.31, 129.99, 129.68, 129.16, 128.23, 127.76, 60.92, 54.98, 44.61, 20.73, 10.25. MS (ESI^+^) *m/z*: 288 [M+H]^+^. Anal. Calc. for C_15_H_21_N_5_O·HCl (323.15): C, 55.64; H, 6.85; N, 21.63; found: C, 55.11; H, 6.62; N, 21.56.

*N-Sec-butyl-4-((4-(hydroxymethyl)-1H-1,2,3-triazol-1-yl)methyl)benzamidine* (**4b**): Yield, 47.4%; white crystals; m.p. 118–120 °C. ^1^H-NMR (400 MHz, MeOD) *δ*: 8.02 (s, 1H, triazole-H), 7.71 (d, *J* = 8.2 Hz, 2H), 7.52 (d, *J* = 8.2 Hz, 2H, benzene-H), 5.72 (s, 2H, CH_2_), 4.65 (s, 2H, CH_2_), 3.81 (dd, *J* = 13.2, 6.6 Hz, 1H, CH), 3.28 (m, 2H, N-CH_2_), 1.62–1.48 (m, 3H, CH_3_), 1.31 (d, *J* = 6.5 Hz, 3H, CH_3_). MS (ESI^+^) *m/z*: 288 [M+H]^+^. Anal. Calc. for C_15_H_21_N_5_O·HCl (323.15): C, 55.64; H, 6.85; N, 21.63; found: C, 55.23; H, 6.71; N, 21.75.

*N,N-Diethyl-4-((4-(hydroxymethyl)-1H-1,2,3-triazol-1-yl)methyl)benzamidine* (**4c**): Yield, 42.1%; yellow crystals; m.p. > 300 °C. ^1^H-NMR (500 MHz, DMSO) *δ*: 8.13 (s, 1H, triazole-H), 8.06 (d, *J* = 8.3 Hz, 2H, benzene-H), 7.46 (d, *J* = 8.3 Hz, 2H, benzene-H), 5.71 (s, 2H, CH_2_), 4.51 (d, *J* = 5.1 Hz, 2H, CH_2_), 3.89 (s, 4H, N-CH_2_). ^13^C-NMR (125 MHz, DMSO) *δ*: 164.49, 148.93, 142.23, 129.28, 128.63, 124.19, 123.73, 55.46, 52.57, 45.94. MS (ESI^+^) *m/z*: 288 [M+H]^+^. Anal. Calc. for C_15_H_21_N_5_O·HCl (323.15): C, 55.64; H, 6.85; N, 21.63; found: C, 55.37; H, 6.65; N, 21.73.

*N-Cyclohexyl-4-((4-(hydroxymethyl)-1H-1,2,3-triazol-1-yl)methyl)benzamidine* (**4d**): Yield, 42.1%; white crystals; m.p. 171–173 °C. ^1^H-NMR (400 MHz, MeOD) *δ*: 8.21 (s, 1H, triazole-H), 7.79 (d, *J* = 8.1, 1.5 Hz, 2H, benzene-H), 7.73 (d, *J* = 8.3 Hz, 2H, benzene-H), 5.77 (s, 2H, CH_2_), 5.35 (s, 2H, CH_2_), 3.82 (d, *J* = 6.7 Hz, 1H, N-CH), 1.79–1.65 (m, 4H, CH_2_), 1.65–1.52 (m, 2H, CH_2_), 1.34 (d, *J* = 6.5 Hz, 3H, CH_2_), 1.04 (t, *J* = 7.3 Hz, 3H, CH_2_). MS (ESI^+^) *m/z*: 314 [M+H]^+^. Anal. Calc. for C_17_H_23_N_5_O·HCl (349.17): C, 58.36; H, 6.91; N, 20.02; found: C, 57.97; H, 6.85; N, 20.34.

*N,N-Dibutyl-4-((4-(hydroxymethyl)-1H-1,2,3-triazol-1-yl)methyl)benzamidine* (**4e**): Yield, 42.1%; white crystals; m.p. 111–112 °C. ^1^H-NMR (400 MHz, MeOD) *δ*: 7.99 (s, 1H, triazole-H), 7.87–7.82 (m, 2H, benzene-H), 7.47–7.43 (m, 2H, benzene-H), 5.69 (s, 2H, CH_2_), 5.65 (s, 2H, CH_2_), 2.81–2.74 (m, 4H, N-CH_2_), 1.80 (d, *J* = 7.5 Hz, 4H, CH_2_), 1.24 (m, 4H, CH_2_), 0.80 (t, *J* = 7.3 Hz, 6H, CH_3_). MS (ESI^+^) *m/z*: 344 [M+H]^+^. Anal. Calc. for C_19_H_29_N_5_O·HCl (379.21): C, 60.06; H, 7.96; N, 18.43; found: C, 59.78; H, 7.81; N, 18.71.

### 3.5. Synthesis of 1-Nitro-2-(prop-2-ynyloxy) Benzene (**7**)

2-Nitrophenol (**5**, 9.10 g, 65 mmol) and K_2_CO_3_ (9.0 g, 65 mmol) were dissolved in acetone (60 mL) in a three necked flask equipped with a thermometer. Then a solution of propargyl bromide (16.03 g, 300 mmol) in acetone (30 mL) was added dropwise at ambient temperature in about 15 min while stirring. Then the reaction mixture was heated to reflux for 3 h after being stirred for 30 min when TLC (petroleum ether/ethyl acetate = 6:1) indicated no **5** remained. The mixture was allowed to cool to ambient temperature and the resulting white solid was recrystallized in ethanol, to afford yellowish needle-shaped crystals of **7** (8.11 g, 70%); m.p. 115–116 °C. ^1^H-NMR (400 MHz, CDCl_3_) *δ*: 7.85 (dd, *J* = 8.1, 1H, benzene-H), 7.64–7.51 (m, 1H, benzene-H), 7.27 (t, *J* = 8.8 Hz, 1H, benzene-H), 7.10 (t, *J* = 7.8 Hz, 1H, benzene-H), 4.85 (d, *J* = 2.4 Hz, 2H, CH_2_), 2.59 (t, *J* = 2.4 Hz, 1H CH). ^13^C-NMR (100 MHz, CDCl_3_) *δ*: 150.76, 140.35, 134.02, 125.76, 121.45, 115.49, 77.42, 76.78, 57.20.

### 3.6. Synthesis of 4-((4-((2-Nitrophenoxy)methyl)-1H-1,2,3-triazol-1-yl)methyl) Benzonitrile (**8**)

A solution (260 mL) of **3** prepared from 4-cyanobenzyl bromide (39.20 g, 200 mmol) was added to a 500-mL round bottom flask, followed by the evaporation of the solvent under reduced pressure. 1-Nitro-2-(prop-2-ynyloxy) benzene (**7**, 35.4 g, 200 mmol) was dissolved in *t*-BuOH (300 mL). The solution of **7** and distilled water (100 mL) was mixed with the residue of **3** in the flask and followed by the addition of sodium ascorbate (250 mg, 1.26 mmol, in 3 mL of water) and cupric sulfate (CuSO_4_·5H_2_O, 85 mg, 0.34 mmol, in 2 mL of water). After stirring at 60 °C for 24 h, the mixture was allowed to cool to room temperature. Compound **8** (58.21 g, 82%) was obtained as a white solid after the evaporation of about half of the solvent, filtration and drying at 110 °C, m.p. 136–138 °C. ^1^H-NMR (500 MHz, DMSO) *δ*: 8.36 (s, 1H, triazole-H), 7.86 (m, 2H, benzene-H), 7.67 (t, *J* = 7.4 Hz, 1H, benzene-H), 7.58 (d, *J* = 8.4 Hz, 1H, benzene-H), 7.46 (d, *J* = 8.1 Hz, 2H, benzene-H), 7.15 (t, *J* = 7.7 Hz, 1H, benzene-H), 5.77 (s, 2H, CH_2_), 5.38 (s, 2H, CH_2_). ^13^C-NMR (125 MHz, DMSO) *δ*: 151.01, 142.73, 141.86, 140.31, 134.76, 133.21, 129.16, 125.85, 125.40, 121.50, 118.99, 116.21, 111.45, 63.08, 52.70.

### 3.7. General Procedure for the Synthesis of 4-((4-((2-Nitrophenoxy)methyl)-1H-1,2,3 -triazol-1-yl)-methyl)-benzamidines **9a**–**e**

A suspension of **8** (3.55 g, 10 mmol), in absolute ethanol (30 mL) was saturated with anhydrous HCl under water-ice bath cooling; the solution was then stirred at room temperature for 4–5 days. After evaporating most of the solvent, the residue was diluted with dry ether, and the resultant solid obtained by filtration was dissolved in dry methanol. The corresponding amines (40 mmol) were added to the solution and stirred for 24 h under anhydrous conditions. Then amidines **9a**–**d** were treated with the same manner described for compound **4**. Compounds **9** were purified by column chromatography on silica gel, using dichloromethane/methanol (7:1, v/v) as the eluent.

*4-((4-((2-Nitrophenoxy)methyl)-1H-1,2,3-triazol-1-yl)methyl)-N-butylbenzamidine* (**9a**): Yield, 51.2%; yellow crystals; m.p. 119–121 °C. ^1^H-NMR (400 MHz, MeOD) *δ*: 8.25 (s, 1H, triazole-H), 7.77 (d, *J* = 8.3 Hz, 1H, benzene-H), 7.75 (d, *J* = 8.3 Hz, 2H, benzene-H), 7.65–7.60 (m, 1H, benzene-H), 7.60–7.56 (m, 1H, benzene-H), 7.53 (d, *J* = 8.2 Hz, 2H, benzene-H), 7.11 (t, *J* = 7.7 Hz, 1H, benzene-H) , 5.77 (s, 2H, CH_2_), 5.34 (s, 2H, CH_2_), 3.46 (t, J = 7.2 Hz, 2H, N-CH_2_), 1.74 (m, 2H, CH_2_), 1.48 (m, 2H, CH_2_), 1.01 (t, J = 7.3 Hz, 3H, CH_2_). ^13^C-NMR (100 MHz, MeOD) *δ*: 166.02, 153.22, 143.27, 142.65, 131.41, 130.87, 130.73, 130.68, 130.49, 127.22, 127.06, 123.17, 117.67, 64.74, 55.05, 41.37, 31.38, 21.51, 14.75. MS (ESI^+^) *m/z*: 409 [M+H]^+^. Anal. Calc. for C_21_H_24_N_6_O_3_·HCl (444.17): C, 56.69; H, 5.66; N, 18.89; found: C, 56.78; H, 5.81; N, 18.81. 

*4-((4-((2-Nitrophenoxy)methyl)-1H-1,2,3-triazol-1-yl)methyl)-N-isobutylbenzamidine* (**9b**): Yield, 36.5%; white solid; m.p. 109–110 °C. ^1^H-NMR (400 MHz, MeOD) *δ*: 8.22 (s, 1H, triazole-H), 7.79–7.74 (m, 1H, benzene-H), 7.73 (d, *J* = 8.2 Hz, 2H, benzene-H), 7.60 (t, *J* = 7.3 Hz, 1H, benzene-H), 7.52 (d, *J* = 8.2 Hz, 2H, benzene-H), 7.09 (t, *J* = 7.6 Hz, 1H, benzene-H), 5.75 (s, 2H, CH_2_), 5.32 (s, 2H, CH_2_), 3.30–3.22 (d, 2H, N-CH_2_), 2.06 (m, 1H, CH), 1.02 (d, *J* = 6.9 Hz, 6H, CH_3_). ^13^C-NMR (100 MHz, MeOD) *δ*: 166.36, 153.23, 143.28, 143.31, 136.12, 131.53, 130.88, 130.72, 130.48, 127.15, 127.06, 123.17, 117.66, 64.76, 55.06, 52.30, 29.43, 21.25. MS (ESI^+^) *m/z*: 409 [M+H]^+^. Anal. Calc. for C_21_H_24_N_6_O_3_·HCl (444.17): C, 56.69; H, 5.66; N, 18.89; found: C, 56.73; H, 5.71; N, 18.82. 

*4-((4-((2-Nitrophenoxy)methyl)-1H-1,2,3-triazol-1-yl)methyl)-N,N-diethylbenzamidine* (**9c**): Yield, 45.2%; white solid; m.p. 135–137 °C. ^1^H-NMR (400 MHz, MeOD) *δ*: 8.21 (s, 1H, triazole-H), 7.76–7.73 (m, 1H, benzene-H), 7.71 (d, *J* = 8.2 Hz, 2H, benzene-H), 7.60 (t, *J* = 7.3 Hz, 1H, benzene-H), 7.52 (d, *J* = 8.2 Hz, 2H, benzene-H), 7.45 (d, *J* = 8.4 Hz, 1H, benzene-H), 7.10 (t, *J* = 7.6 Hz, 1H, benzene-H), 5.74 (s, 2H, CH_2_), 5.31 (s, 2H, CH_2_), 3.09 (q, J = 7.3 Hz, 4H, N-CH_2_), 1.31 (t, J = 7.3 Hz, 6H, CH_3_). ^13^C-NMR (100 MHz, MeOD) *δ*: 166.33, 153.20, 143.99, 142.71, 136.11, 130.71, 130.52, 130.18, 127.04, 123.18, 117.75, 64.76, 55.23, 44.41, 12.47. MS (ESI^+^) *m/z*: 409 [M+H]^+^. Anal. Calc. for C_21_H_24_N_6_O_3_·HCl (444.17): C, 56.69; H, 5.66; N, 18.89; found: C, 56.81; H, 5.69; N, 18.78.

*4-((4-((2-Nitrophenoxy)methyl)-1H-1,2,3-triazol-1-yl)methyl)-N-cyclohexylbenzamidine* (**9d**): Yield, 57.0%; white crystals; m.p. 110–113 °C. ^1^H-NMR (400 MHz, MeOD) *δ*: 8.17 (s, 1H, triazole-H), 7.77–7.71 (m, 1H, benzene-H), 7.67 (d, *J* = 8.2 Hz, 2H, benzene-H), 7.60–7.53 (m, 1H, benzene-H), 7.48 (d, *J* = 8.2 Hz, 2H, benzene-H), 7.42 (d, *J* = 8.4 Hz, 1H, benzene-H), 7.07 (t, *J* = 7.6 Hz, 1H, benzene-H), 5.72 (s, 2H, CH_2_), 5.30 (s, 2H, CH_2_), 3.70–3.59 (m, 1H, N-CH), 1.51–1.35 (m, 5H, CH_2_), 1.16 (m, 5H, CH_2_). ^13^C-NMR (100 MHz, MeOD) *δ*: 165.09, 153.26, 145.33, 143.21, 136.09, 131.79, 131.45, 130.63, 130.59, 127.07, 123.18, 117.64, 64.79, 55.07, 52.41, 32.76, 26.62, 26.21. MS (ESI^+^) *m/z*: 435 [M+H]^+^. Anal. Calc. for C_19_H_29_N_5_O·HCl (470.18): C, 58.66; H, 5.78; Cl, 7.53; N, 17.84; found: C, 58.78; H, 5.80; N, 17.91.

*4-((4-((2-Nitrophenoxy)methyl)-1H-1,2,3-triazol-1-yl)methyl)-N,N-dibutylbenzamidine* (**9e**): Yield, 54.6%; light yellow crystals; m.p. 119–121 °C. ^1^H-NMR (400 MHz, MeOD) *δ*: 8.10 (s, 1H, triazole-H), 7.82 (d, *J* = 8.2 Hz, 2H, benzene-H), 7.77 (m, 1H, benzene-H), 7.57 (t, *J* = 7.4 Hz, 1H, benzene-H), 7.41 (d, *J* = 8.5 Hz, 1H, benzene-H), 7.34 (d, *J* = 8.2 Hz, 2H, benzene-H), 7.08 (t, *J* = 7.7 Hz, 1H, benzene-H), 5.67 (s, 2H, CH_2_), 5.32 (s, 2H, CH_2_), 4.23 (t, *J* = 6.9 Hz, 4H, N-CH_2_), 3.59 (m, 4H, CH_2_), 1.48–1.29 (m, 4H, CH_2_), 0.97 (t, *J* = 7.4 Hz, 6H, CH_3_). ^13^C-NMR (100 MHz, MeOD) *δ*:165.09, 153.01, 145.11, 143.44, 135.75, 130.70, 129.83, 129.58, 126.91, 122.98, 117.44, 64.60, 55.01, 52.25, 29.98, 21.43, 19.98. MS (ESI^+^) *m/z*: 465 [M+H]^+^. Anal. Calc. for C_19_H_29_N_5_O·HCl (500.23): C, 59.93; H, 6.64; N, 16.77; found: C, 59. 87; H, 6.73; N, 16.72.

### 3.8. Synthesis of 1-Nitro-4-(prop-2-ynyloxy)benzene (**12**) [51]

Compound **12** was prepared following the same procedure used for **7**. Yield, 76%; m.p. 118–119 °C; ^1^H-NMR (400 MHz, CDCl_3_) *δ*: 8.22 (d, *J* = 9.3 Hz, 2H, benzene-H), 7.06 (d, *J* = 9.3 Hz, 2H, benzene-H), 4.81 (d, *J* = 2.4 Hz, 2H, CH_2_), 2.60 (s, 1H, CH). ^13^C-NMR (100 MHz, CDCl_3_) *δ*: 162.35, 125.85, 114.99, 77.42, 77.10, 76.80, 56.31.

### 3.9. Synthesis of 3-(Azidomethyl)benzonitrile (**14**)

3-(Bromomethyl)benzonitrile (**13**, 39.2 g, 200 mmol) was dissolved in DMF (180 mL) in an Erlenmeyer flask equipped with a thermometer, then NaN_3_ (16.50 g, 250 mmol, in 90 mL of distilled water) was added in portions under water-ice bath cooling while stirring, keeping the mixture under 10 °C. After the addition of the azide, the mixture was stirred for 1.5 h at 30 °C when the color of the reaction mixture turned colorless and TLC indicated no **13** remained. The mixture was diluted with toluene (180 mL) and distilled water (500 mL) in turn. The aqueous phase was extracted with toluene (2 × 60 mL) and the organic phase was combined and dried with MgSO_4_. After removing of the solvent, **14** (39.6 g, 97%) was obtained as a white solid; m.p. 30–31 °C. ^1^H-NMR (500 MHz, CDCl_3_) *δ*: 7.62–7.57 (m, 2H, benzene-H), 7.57–7.54 (m, 1H, benzene-H), 7.51–7.45 (m, 1H, benzene-H), 4.41 (s, 2H, CH_2_). ^13^C-NMR (125 MHz, CDCl_3_) *δ*: 137.25, 132.41, 131.80, 131.38, 129.73, 118.49, 112.85, 53.62.

### 3.10. General Procedure for the Synthesis of 3-(Azidomethyl)benzamidines **15a**–**d** [42]

Absolute ethanol (80 mL) was added to the solution (25 mL) of **14**. The mixture was sealed and stirred for 7 days at room temprature after being saturated with anhydrous HCl at ice-water bath. The residue was recrystallized from ether, and then dissolved in absolute methanol (30 mL). The solution was stirred at 30 °C overnight after the corresponding amines (43 mmol) were added. After evaporating most of the solvent, the residue was mixed with ethanol (5 mL) and 2 N aqueous NaOH (5 mL). Ether (50 mL) was added after the aqueous phase being removed, and the target amidines **15a**–**d** was obtained by precipitation. 

*3-(Azidomethyl)-N-butylbenzamidine* (**15a**): Yield, 75.3%; yellow crystals; m.p. 106–109 °C. ^1^H-NMR (400 MHz, MeOD) *δ*: 7.67 (s, 1H, benzene-H), 7.64 (d, *J* = 3.9 Hz, 2H, benzene-H), 7.57 (t, *J* = 7.6 Hz, 1H, benzene-H), 4.48 (s, *J* = 6.3 Hz, 2H, CH_2_), 3.41 (t, *J* = 7.3 Hz, 2H, N-CH_2_), 1.75–1.52 (m, 2H, CH_2_), 1.50–1.30 (m, 2H, CH_2_), 0.94 (t, 7.4 Hz, 3H, CH_3_). ^13^C-NMR (100 MHz, MeOD) *δ*: 164.17, 137.64, 132.78, 129.80, 129.48, 127.23, 127.17, 53.38, 46.98, 29.36, 19.75, 12.50. MS (ESI^+^) *m/z*: 232 [M+H]^+^. Anal. Calc. for C_12_H_1__7_N_5_·HCl (267.13): C, 53.83; H, 6.78; N, 26.16; found: C, 53. 87; H, 6.71; N, 26.18. 

*N-Sec-butyl-3-(azidomethyl)benzamidine* (**15b**): Yield, 71.2%; yellow crystals; m.p. 108–110 °C. ^1^H-NMR (400 MHz, DMSO) *δ*: 9.64 (s, 2H, N-H), 8.07 (s, 1H, benzene-H), 7.98 (d, *J* = 7.7 Hz, 2H, benzene-H), 7.89 (t, *J* = 7.7 Hz, 1H, benzene-H), 4.38 (s, 2H, CH_2_), 3.28 (m, 1H, N-CH), 1.85 (m, 2H, CH_2_), 1.45 (d, *J* = 6.6 Hz, 3H, CH_3_), 1.21 (t, *J* = 7.4 Hz, 3H, CH_3_). ^13^C-NMR (100 MHz, DMSO) *δ*: 162.39, 136.98, 133.17, 130.16, 129.62, 128.65, 128.53, 53.38, 51.12, 28.59, 19.65, 10.83. MS (ESI^+^) *m/z*: 232 [M+H]^+^. Anal. Calc. for C_12_H_1__7_N_5_·HCl (267.13): C, 53.83; H, 6.78; N, 26.16; found: C, 53. 85; H, 6.82; N, 26.19.

*3-(Azidomethyl)-N-cyclohexylbenzamidine* (**15c**): Yield, 78.0%; white crystals; m.p. 110–111 °C. ^1^H-NMR (400 MHz, MeOD) *δ*: 7.77 (s, 1H, benzene-H), 7.59 (d, *J* = 7.7 Hz, 2H, benzene-H), 7.39 (t, *J* = 7.7 Hz, 1H, benzene-H), 4.42 (s, 2H, CH_2_), 2.94–2.79 (m, 1H, N-CH), 1.91 (m, 5H, CH_3_), 1.80–1.65 (m, 5H, CH_3_). ^13^C-NMR (100 MHz, MeOD) *δ*: 163.08, 137.52, 132.67, 131.37, 130.09, 129.41, 127.31, 127.22, 53.39, 48.28, 31.29, 24.80. MS (ESI^+^) *m/z*: 258 [M+H]^+^. Anal. Calc. for C_14_H_19_N_5_·HCl (293.14): C, 57.23; H, 6.86; Cl, 12.07; N, 23.84; found: C, 57. 35; H, 6.81; N, 26.20.

*3-(Azidomethyl)-N,N-diethylbenzamidine* (**15d**): Yield, 55.4%; white crystals; m.p. 99–101 °C. ^1^H-NMR (400 MHz, DMSO) *δ*: 9.44 (s, 3H, N-H), 8.10 (s, 1H, benzene-H), 7.93 (d, *J* = 7.7 Hz, 2H, benzene-H), 7.87 (t, *J* = 7.7 Hz, 1H, benzene-H), 4.45 (s, 2H, CH_2_), 2.82–2.75 (m, 4H, N-CH_2_), 1.16 (t, *J* = 7.4 Hz, 6H, CH_3_). MS (ESI^+^) *m/z*: 232 [M+H]^+^. Anal. Calc. for C_12_H_1__7_N_5_·HCl (267.13): C, 53.83; H, 6.78; N, 26.16; found: C, 53. 86; H, 6.75; N, 26.14.

### 3.11. General Procedure for the Synthesis of 3-((4-(Hydroxymethyl)-1H-1,2,3-triazol-1-yl)methyl)-benzamidines **16a**–**d**

Compound **15** (4.5 mmol) and propargyl alcohol (28 mg, 5 mmol) was dissolved in a mixture of *t*-butanol (30 mL) and distilled water (12 mL), and then, ascorbate sodium(45 mg, 0.23 mmol, in 1 mL water) and CuSO_4_·5H_2_O (10 mg, 0.04 mmol, in 1 mL water) were added to the mixture. After stirring at 60 °C for 48 h, half of the solvent was evaporated and the target molecule was obtained by recrystallization from ether (**16a**) or column chromatography on silica gel (**16b**, **16c**, **16d**), using dichloromethane/methanol (5:1, v/v) as the eluent.

*N-Butyl-3-((4-(hydroxymethyl)-1H-1,2,3-triazol-1-yl)methyl)benzamidine* (**16a**): Yield, 68.8%; light yellow crystals; m.p. 106–109 °C. ^1^H-NMR (400 MHz, MeOD) *δ*: 8.07 (s, 1H, triazole-H), 7.80 (s, 1H, benzene-H), 7.66 (d, *J* = 7.7 Hz, 2H, benzene-H), 7.59 (t, *J* = 7.7 Hz, 1H, benzene-H), 5.72 (s, 2H, CH_2_), 4.65 (s, 2H, CH_2_), 3.46 (t, 2H, N-CH_2_), 1.79–1.68 (m, 2H, CH_2_), 1.48 (m, 2H, CH_2_), 0.99 (m, 3H, CH_3_). ^13^C-NMR (100 MHz, DMSO) *δ*: 163.18, 136.16, 132.01, 129.11, 128.93, 126.94, 126.64, 122.58, 54.22, 51.94, 42.09, 38.35, 18.50, 11.92. MS (ESI^+^) *m/z*: 288 [M+H]^+^. Anal. Calc. for C_15_H_21_N_5_O·HCl (323.15): C, 55.64; H, 6.85; N, 21.63; found: C, 55.41; H, 6.72; N, 21.57. 

*N-Sec-butyl-3-((4-(hydroxymethyl)-1H-1,2,3-triazol-1-yl)methyl)benzamidine* (**16b**): Yield, 71.1%; light yellow crystals; m.p. 108–110 °C. ^1^H-NMR (400 MHz, MeOD) *δ*: 8.05 (s, 1H, triazole-H), 7.72 (s, 1H, benzene-H), 7.67 (d, *J* = 7.7 Hz, 2H, benzene-H), 7.59 (t, *J* = 7.6 Hz, 1H, benzene-H), 5.71 (s, 2H, CH_2_), 4.64 (s, 2H, CH_2_), 3.81 (t, J = 7.4 Hz, 1H, N-H), 1.75–1.64 (m, 2H, CH_2_), 1.32 (m, 2H, CH_2_), 1.01 (t, *J* = 7.4 Hz, 3H, CH_3_). 13C-NMR (100 MHz, DMSO) *δ*: 162.68, 136.11, 131.94, 129.40, 128.89, 126.94, 126.63, 122.54, 54.22, 51.92, 50.56, 26.58, 17.26, 16.06. MS (ESI^+^) *m/z*: 288 [M+H]^+^. Anal. Calc. for C_15_H_21_N_5_O·HCl (323.15): C, 55.64; H, 6.85; N, 21.63; found: C, 55.42; H, 6.78; N, 21.71.

*N-Cyclohexyl-3-((4-(hydroxymethyl)-1H-1,2,3-triazol-1-yl)methyl)benzamidine* (**16c**): Yield, 76.2%; light yellow crystals; m.p. 110–111 °C. ^1^H-NMR (400 MHz, DMSO) *δ*: 7.95 (s, 1H, triazole-H), 7.63 (s, 1H, benzene-H), 7.57 (d, *J* = 7.8 Hz, 2H, benzene-H), 7.49 (t, *J* = 7.6 Hz, 1H, benzene-H), 5.61 (s, 2H, CH_2_), 4.55 (s, 2H, CH_2_), 3.59 (m, 1H, N-H), 1.44–1.29 (m, 5H, CH_2_), 1.24 (m, 5H, CH_2_). ^13^C-NMR (100 MHz, DMSO) *δ*: 162.12, 136.04, 131.92, 129.39, 128.84, 126.73, 126.55, 122.46, 54.22, 52.34, 51.92, 51.88, 46.20, 30.30, 23.98. MS (ESI^+^) *m/z*: 314 [M+H]^+^. Anal. Calc. for C_17_H_23_N_5_O·HCl (349.17): C, 58.36; H, 6.91; N, 20.02; found: C, 58.47; H, 6.89; N, 20.13.

*N,N-Diethyl-3-((4-(hydroxymethyl)-1H-1,2,3-triazol-1-yl)methyl)benzamidine* (**16d**): Yield, 70.3%; light yellow crystals; m.p. 99–101 °C. ^1^H-NMR (400 MHz, MeOD) *δ*: 8.09 (s, 1H, triazole-H), 7.65 (d, *J* = 7.4 Hz, 1H, benzene-H), 7.62 (d, *J* = 8.1 Hz, 1H, benzene-H), 7.55 (m, 2H, benzene-H), 5.74 (s, 2H, CH_2_), 4.66 (s, 2H, CH_2_), 3.33–3.27 (m, 4H, N-CH_2_), 1.37 (t, *J* = 7.2 Hz, 6H, CH_3_). ^13^C-NMR (100 MHz, DMSO) *δ*: 163.75, 162.39, 136.43, 130.50, 129.51, 129.04, 126.35, 125.91, 54.24, 51.92, 42.31, 11.63. MS (ESI^+^) *m/z*: 288 [M+H]^+^. Anal. Calc. for C_15_H_21_N_5_O·HCl (323.15): C, 55.64; H, 6.85; N, 21.63; found: C, 55.42; H, 6.78; N, 21.71.

### 3.12. General Procedure for the Synthesis of 3-((4-((4-Nitrophenoxy)methyl)-1H-1,2,3-triazol-1-yl) methyl)benzamidines **17a**–**d**

Compounds **17a**–**d** were prepared using the same procedure as for compounds **16a**–**d**, by replacing the propargyl alcohol with 1-nitro-4-(prop-2-ynyloxy)benzene (**12**).

*3-((4-((4-Nitrophenoxy)methyl)-1H-1,2,3-triazol-1-yl)methyl)-N-butylbenzamidine* (**17a**): Yield, 80.1%; light yellow crystals; m.p. 164–166 °C. ^1^H-NMR (400 MHz, MeOD) *δ*: 8.29 (s, 1H, triazole-H), 8.13 (s, *J* = 9.1 Hz, 2H, benzene-H), 7.74 (s, 1H, benzene-H), 7.67 (d, *J* = 7.1 Hz, 1H, benzene-H), 7.65 (d, *J* = 7.2 Hz, 1H, benzene-H), 7.61 (s, 1H, benzene-H), 7.11 (d, *J* = 9.1 Hz, 2H, benzene-H), 5.71 (s, 2H, CH_2_), 5.24 (s, 2H, CH_2_), 2.93–2.81 (m, 2H, N-CH_2_), 1.74–1.63 (m, 2H, CH_2_), 1.34 (m, 2H, CH_2_), 0.93 (d, *J* = 2.3 Hz, 3H, CH_3_). ^13^C-NMR (100 MHz, DMSO) *δ*: 163.11, 162.58, 140.85, 135.89, 132.13, 132.02, 129.12, 129.00, 128.73, 127.04, 126.77, 126.48, 126.40, 124.18, 114.04, 60.80, 52.10, 42.11, 28.56, 18.95, 11.92. MS (ESI^+^) *m/z*: 409 [M+H]^+^. Anal. Calc. for C_21_H_24_N_6_O_3_·HCl (444.17): C, 56.69; H, 5.66; N, 18.89; found: C, 56.71; H, 5.72; N, 18.84.

*3-((4-((4-Nitrophenoxy)methyl)-1H-1,2,3-triazol-1-yl)methyl)-N-sec-butylbenzamidine* (**17b**): Yield, 69.6%; yellow solid; m.p. 92–94 °C. ^1^H-NMR (400 MHz, MeOD) *δ*: 8.31 (s, 1H, triazole-H), 8.17 (d, *J* = 9.1 Hz, 2H, benzene-H), 7.76 (s, 1H, benzene-H), 7.68 (d, *J* = 7.1 Hz, 1H, benzene-H), 7.66 (d, *J* = 7.2 Hz, 1H, benzene-H), 7.60 (m, 1H, benzene-H), 7.15 (d, *J* = 9.2 Hz, 2H, benzene-H), 5.75 (s, 2H, CH_2_), 5.28 (s, 2H, CH_2_), 3.89–3.75 (m, 1H, N-CH), 1.67 (m, 2H, CH_2_), 1.27 (d, *J* = 6.6 Hz, 3H, CH_3_), 1.02–0.98 (m, 3H, CH_3_). ^13^C-NMR (100 MHz, DMSO) *δ*: 162.64, 162.57, 140.89, 135.89, 132.04, 131.92, 129.43, 128.94, 127.03, 126.77, 126.48, 126.40, 124.08, 113.99, 60.79, 52.05, 50.57, 28.98, 17.25, 8.61. MS (ESI^+^) *m/z*: 409 [M+H]^+^. Anal. Calc. for C_21_H_24_N_6_O_3_·HCl (444.17): C, 56.69; H, 5.66; N, 18.89; found: C, 56.63; H, 5.61; N, 18.93.

*3-((4-((4-Nitrophenoxy)methyl)-1H-1,2,3-triazol-1-yl)methyl)-N-cyclohexylbenzamidine* (**17c**): Yield, 71.5%; brown solid; m.p. 101–103 °C. ^1^H-NMR (400 MHz, MeOD) *δ*: 8.19 (s, 1H, triazole-H), 8.05 (d, *J* = 7.8 Hz, 2H, benzene-H), 7.65 (s, 1H, benzene-H), 7.58 (d, *J* = 7.1 Hz, 1H, benzene-H), 7.56 (d, *J* = 7.1 Hz, 1H, benzene-H), 7.48 (d, *J* = 7.8 Hz, 1H, benzene-H), 7.04 (d, *J* = 7.8 Hz, 2H, benzene-H), 5.63 (s, 2H, CH_2_), 5.17 (s, 2H, CH_2_), 3.18 (m, 1H, N-CH), 1.55 (m, 5H, CH_2_), 1.32 (d, *J* = 10.0 Hz, 5H, CH_2_). MS (ESI^+^) *m/z*: 435 [M+H]^+^. Anal. Calc. for C_19_H_29_N_5_O·HCl (470.18): C, 58.66; H, 5.78; Cl, 7.53; N, 17.84; found: C, 58.78; H, 5.82; N, 17.81.

*3-((4-((4-Nitrophenoxy)methyl)-1H-1,2,3-triazol-1-yl)methyl)-N,N-diethylbenzamidine* (**17d**): Yield, 68.9%; brown solid; m.p. 159–161 °C. ^1^H-NMR (400 MHz, MeOD) *δ*: 8.36 (s, 1H, triazole-H), 8.21 (d, *J* = 8.9 Hz, 2H, benzene-H), 7.68 (s, 1H, benzene-H), 7.64 (m, 2H, benzene-H), 7.54 (d, *J* = 7.7 Hz, 1H, benzene-H), 7.20 (d, *J* = 8.9 Hz, 2H, benzene-H), 5.79 (s, 2H, CH_2_), 5.32 (s, 2H, CH_2_), 3.40–3.25 (m, 4H, N-CH_2_), 1.38 (t, *J* = 7.2 Hz, 6H, CH_3_). MS (ESI^+^) *m/z*: 409 [M+H]^+^. Anal. Calc. for C_21_H_2__4_N_6_O_3_·HCl (444.17): C, 56.69; H, 5.66; N, 18.89; found: C, 56.72; H, 5.61; N, 19.02.

### 3.13. Fungicidal Activity Bioassay: Effect of the New Compounds on the Mycelial Growth of C. lagenarium and B. cinerea in Solid Media

The compounds **4a**–**e**, **9a**–**e**, **15a**–**d**, **16a**–**d**, **17a**–**d** were dissolved in ethanol (5,000 μg/mL) and mixed with sterile molten potato dextrose agar (PDA) to obtain final concentrations of 200 μg/mL. The commercial fungicide carbendazim SC (with a 50% composition) was used as the positive control. Fungicidal activities of the compounds against *B. cinerea* were evaluated *in vitro* using the method given in [52]. The inhibition rate was calculated according to Equation (1):

I_1_ = (D_1_−D_0_)/D_1_ × 100% (1)

where I_1_ is the inhibition rate, D_1_ is the average diameter of mycelia in the blank test, and D_0_ is the average diameter of mycelia in the presence of compounds. The results are given in Table 1.

### 3.14. Effect on C. lagenarium and B. cinerea Activity on Cucumber Leaves [53]

Fungicidal activity against *Colletotrichum lagenarium* and *Botrytis cinerea* was tested on cucumber (*Cucumis sarivus* L.) seedlings at three leaf stages. The commercial fungicide carbendazim SC (with a 50% composition) was adopted as the positive control. Methanol solutions of the compounds and carbendazim (5,000 μg/mL) were diluted to concentrations at 300 μg/mL with 0.1% PEG400 solution of water to obtain solutions that were spread on the surface of the cucumber leaves. After air drying for 24 h, the upper sides of the leaves were inoculated with 4 mm plugs of *C. lagenarium* or *B. cinerea* maintained on PDA. Nine replicates were performed. The inhibition rate was calculated according to Equation (1), and the results are shown in Table 1.

## 4. Conclusions

Eighteen novel benzamidines with 1,2,3-triazole moieties connected to the benzene ring and four benzamidines containing azide groups were synthesized and characterized. Bioassays showed that the synthesized benzamidine compounds had weak antifungal activities against the tested fungi *in vitro*, however, the activities *in vivo* of some compounds to the same strains were excellent. Compounds **4c**, **9a**, **9b**, **16d** and **15b** exhibited significant activities toward *C. lagenarium in vivo*. Compounds **4b**, **4e**, and **17a** and **15b** showed excellent inhibitory activities toward *B. cinerea*.

## Supplementary Materials

Supplementary materials can be accessed at: http://www.mdpi.com/1420-3049/19/5/5674/s1.
