# Supplementary material for: Synthesis and Antifungal Activity of Benzamidine Derivatives Carrying 1,2,3-Triazole Moieties
Source: Molecules. 2014 May 2;19(5):5674–91. doi: 10.3390/molecules19055674 (PMC6270668; doi:10.3390/molecules19055674)

Supplementary Materials

^{1}H-NMR spectrum of compound 2

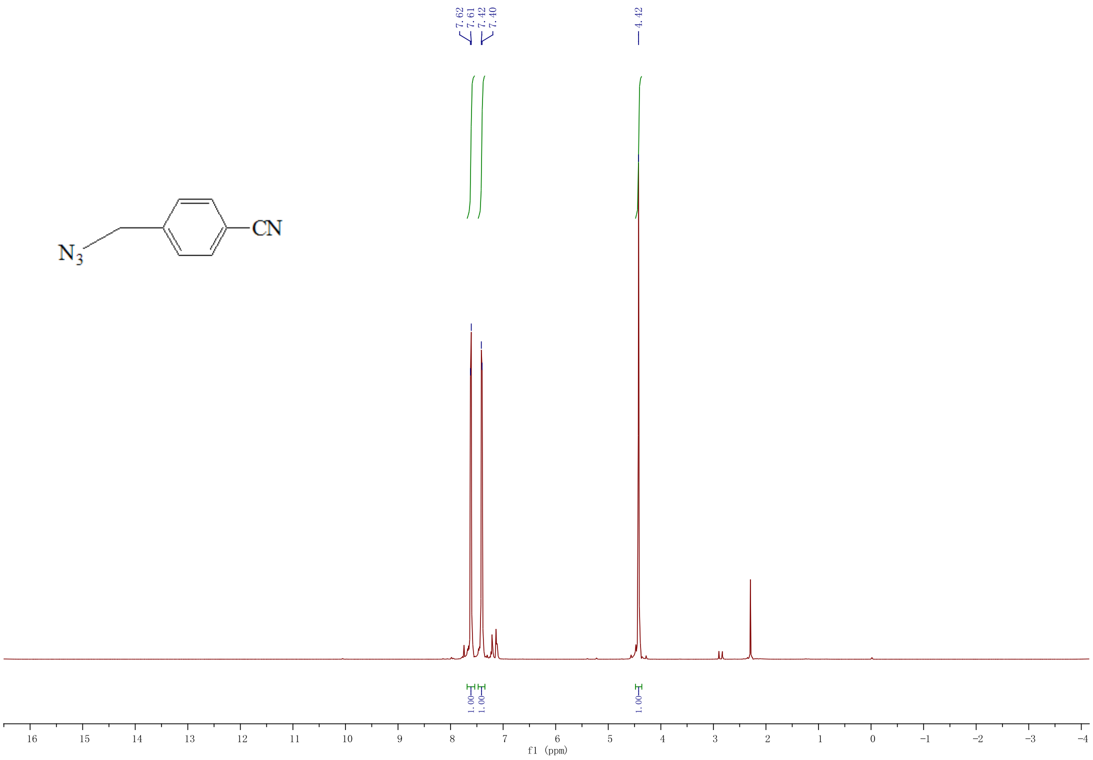

^{13}C-NMR spectrum of compound 2

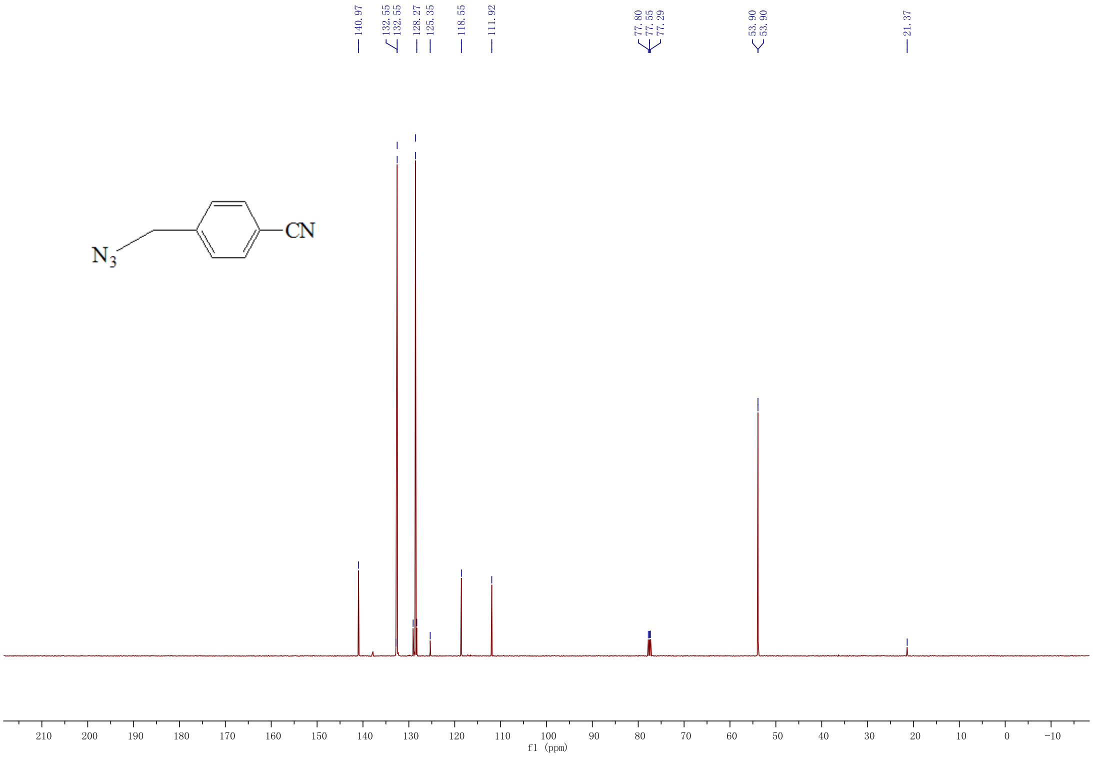

^{1}H-NMR spectrum of compound 3

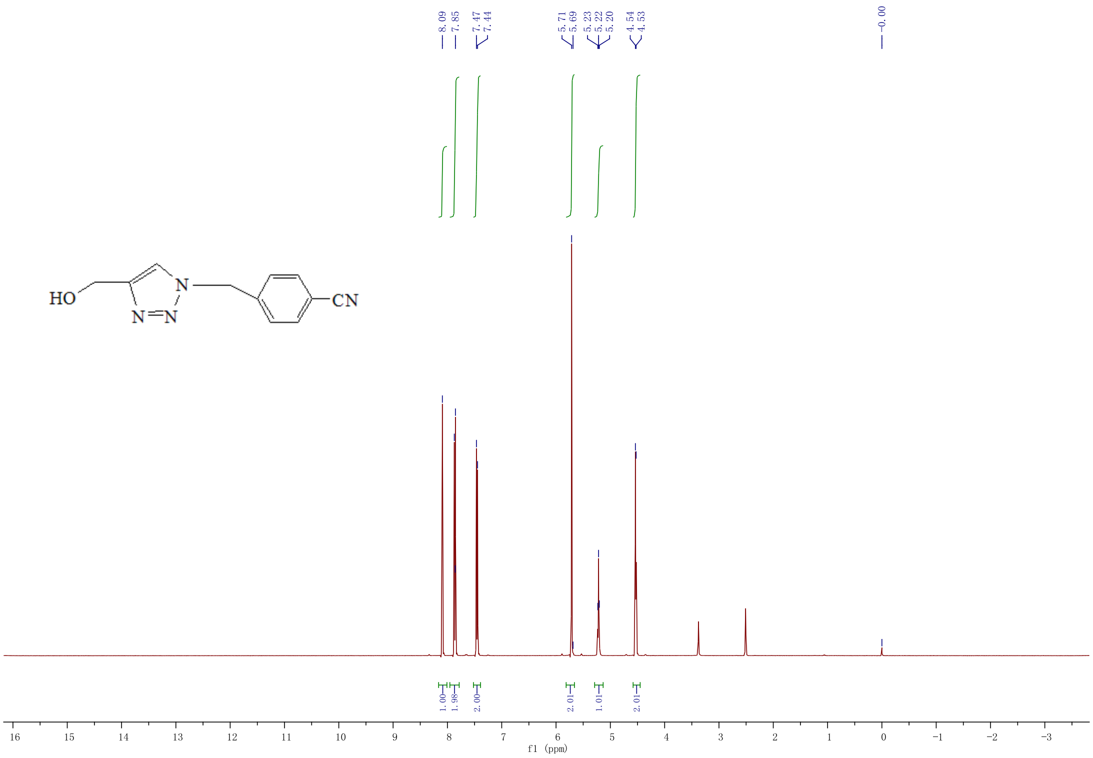

^{13}C-NMR spectrum of compound 3

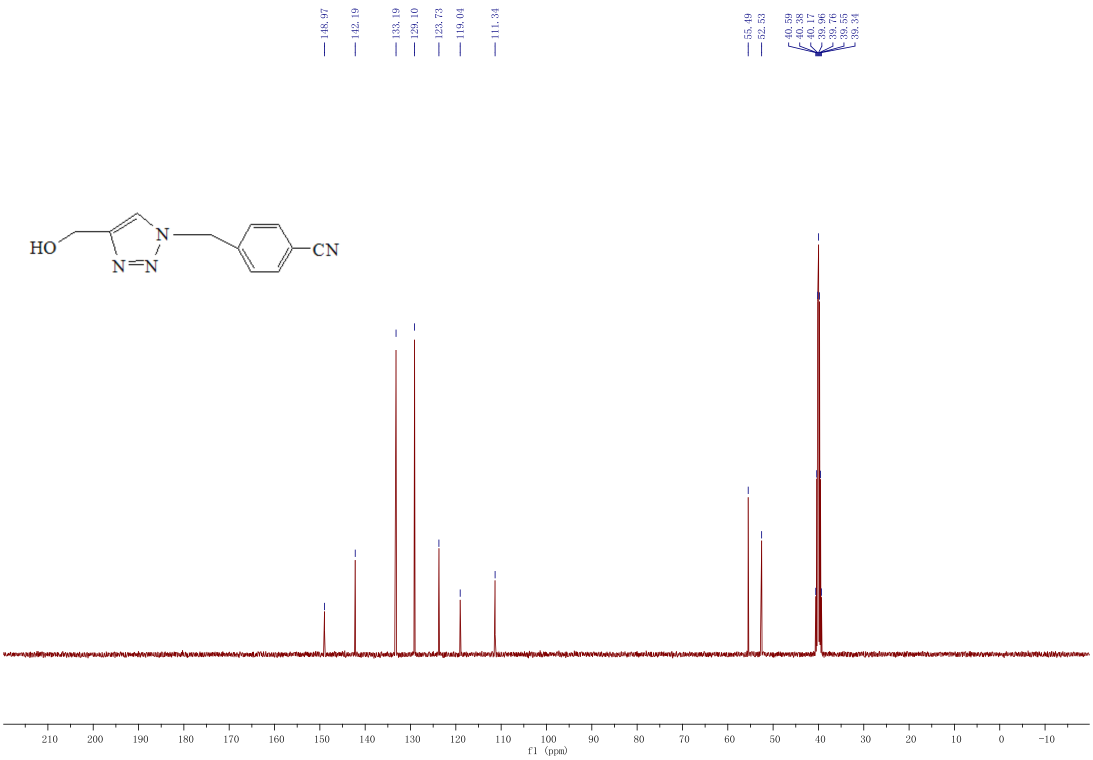

^{1}H-NMR spectrum of compound 4a

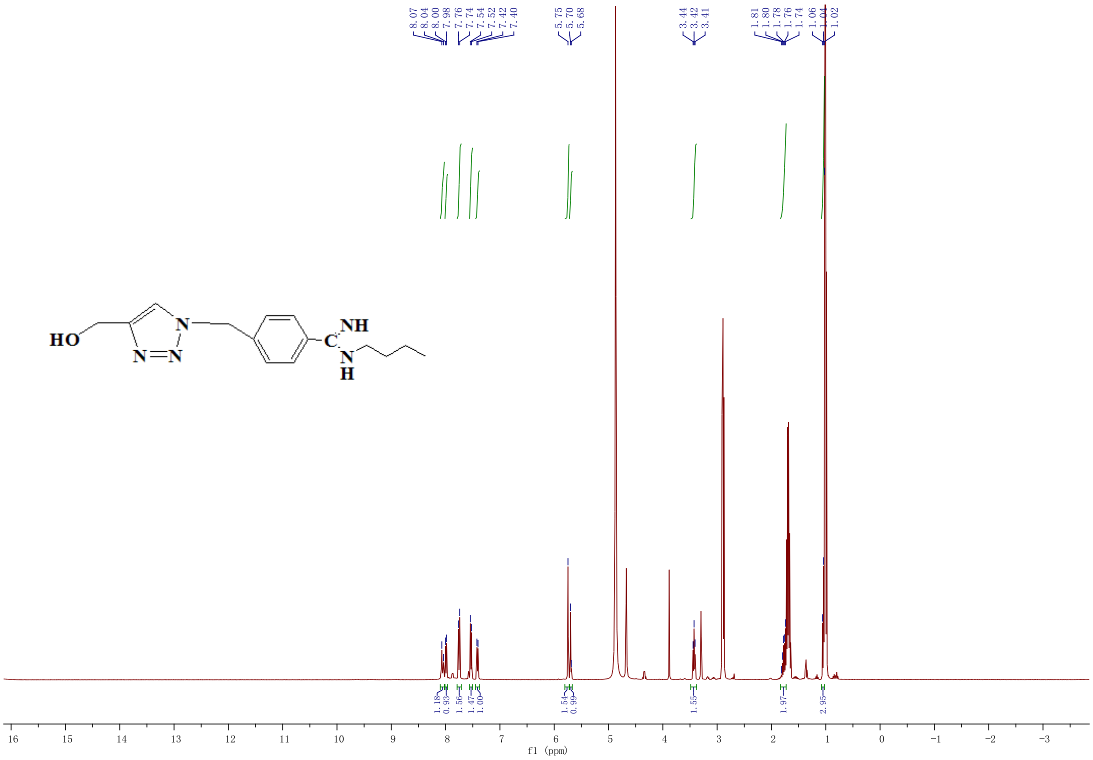

^{1}H-NMR spectrum of compound 4b

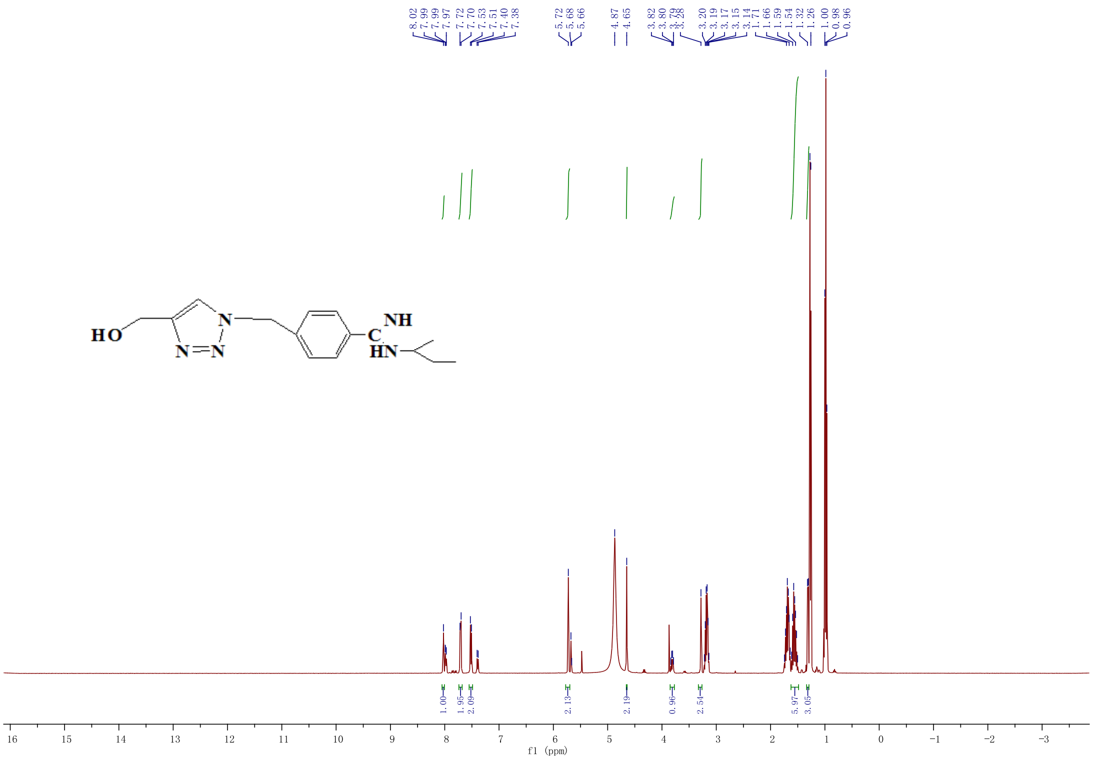

^{1}H-NMR spectrum of compound 7

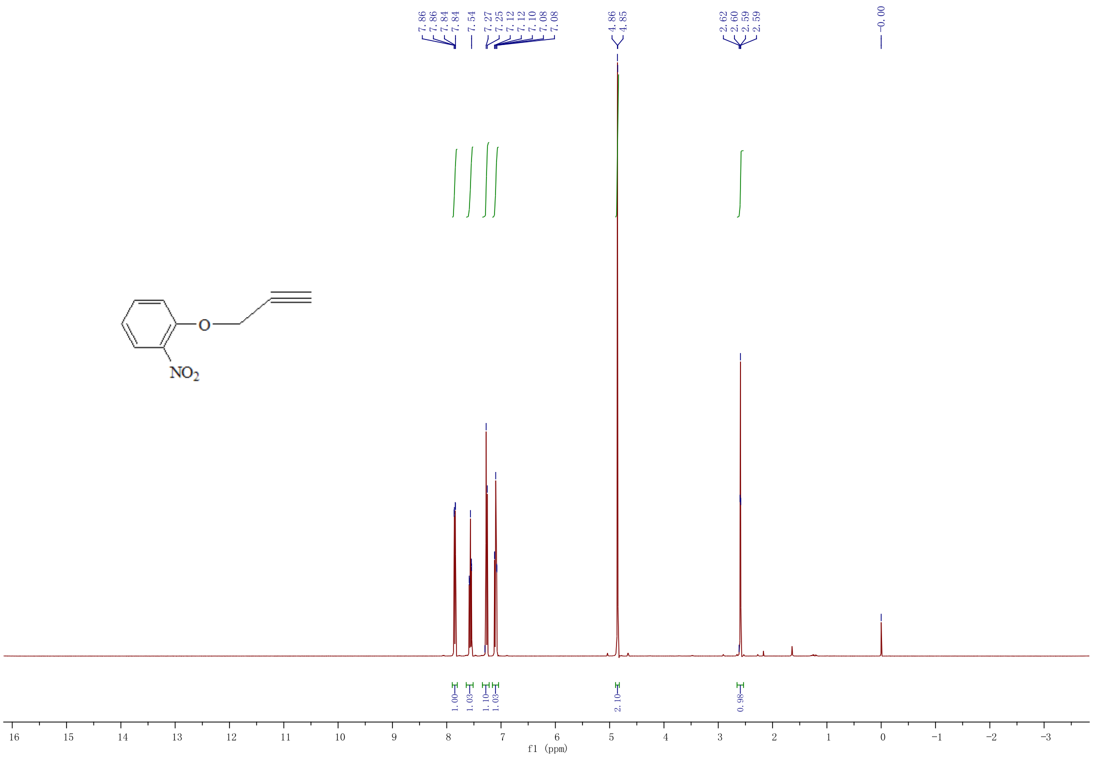

^{13}C-NMR spectrum of compound 7

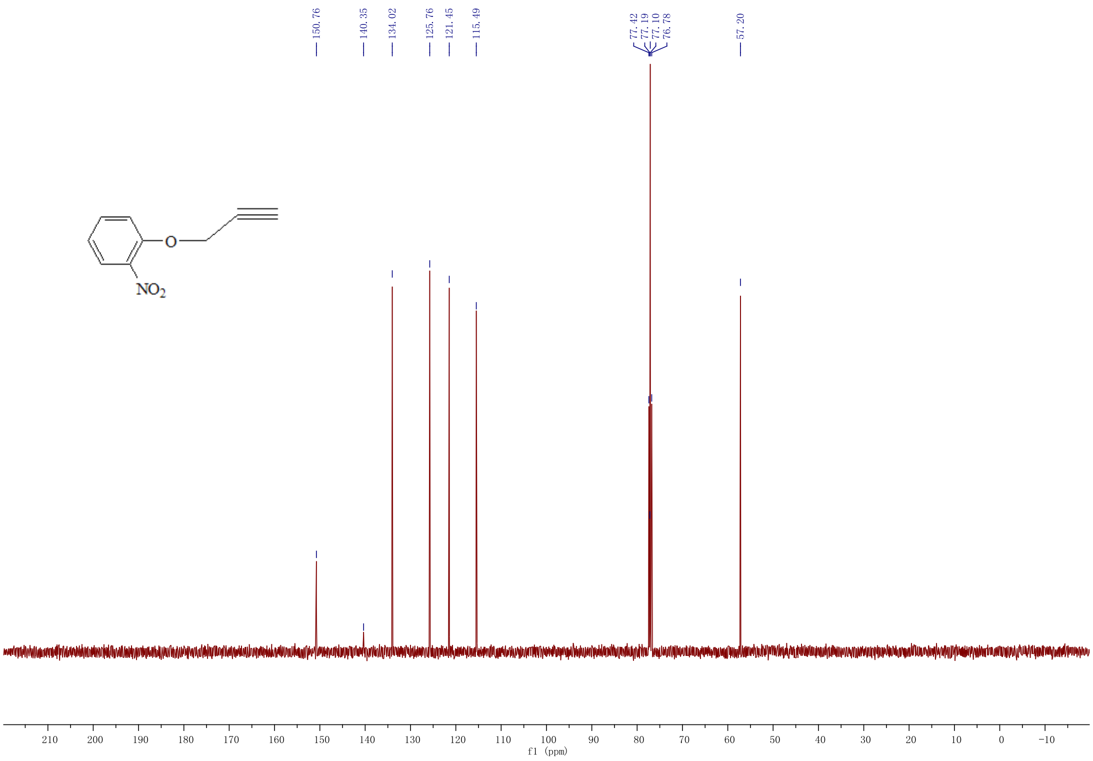

^{1}H-NMR spectrum of compound 8

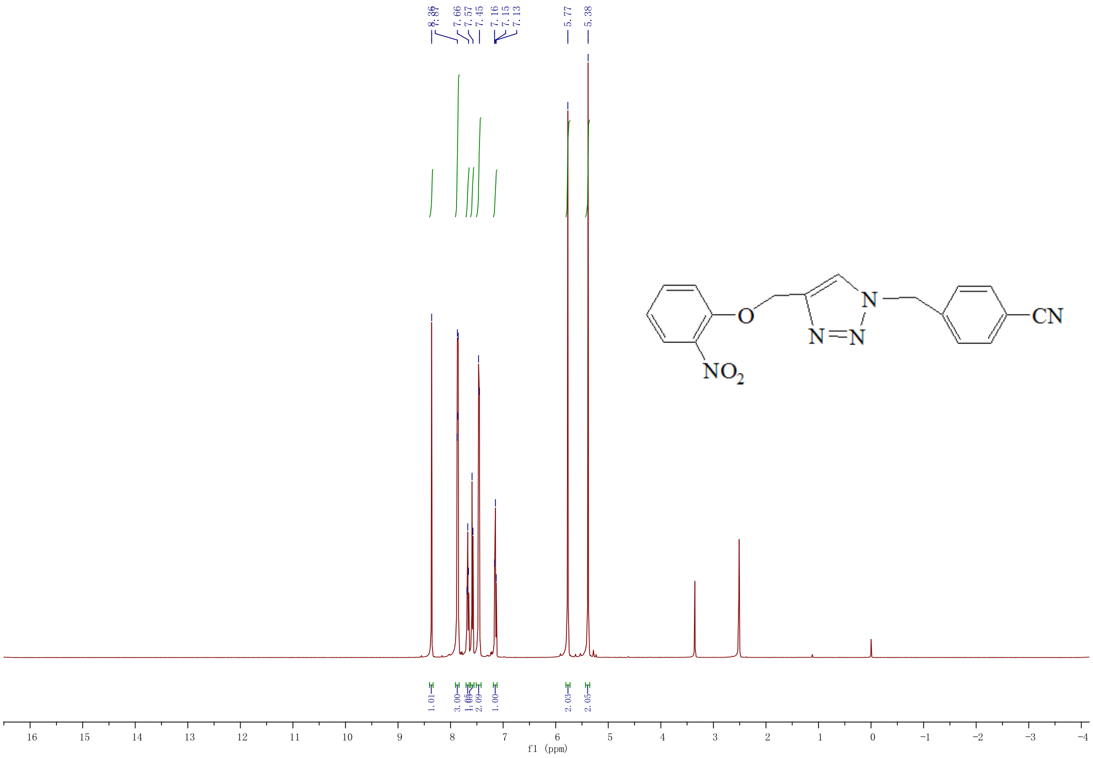

^{13}C-NMR spectrum of compound 8

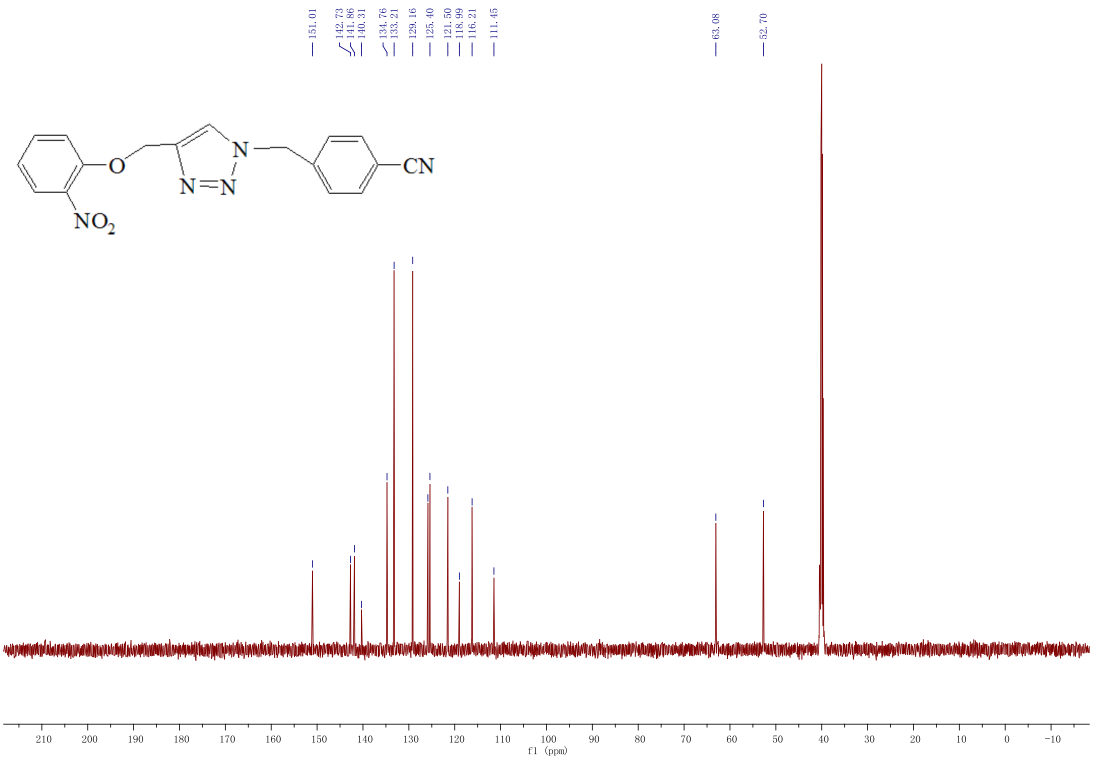

^{1}H-NMR spectrum of compound 9a

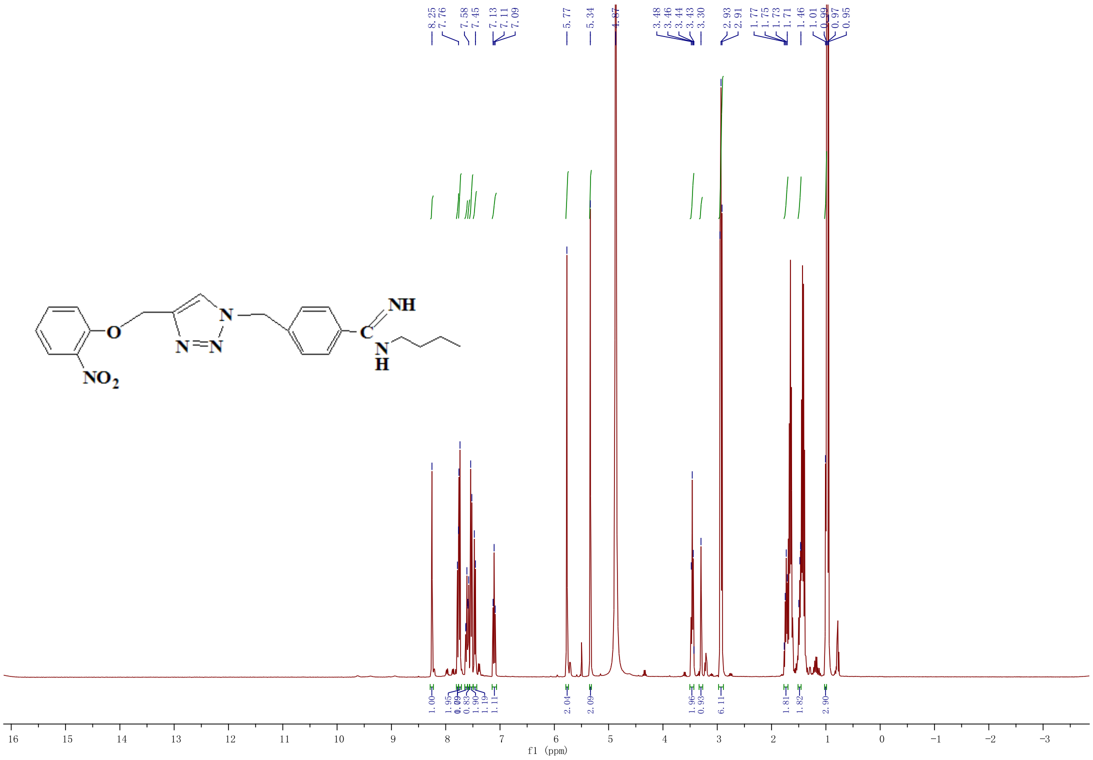

^{1}H-NMR spectrum of compound 9b

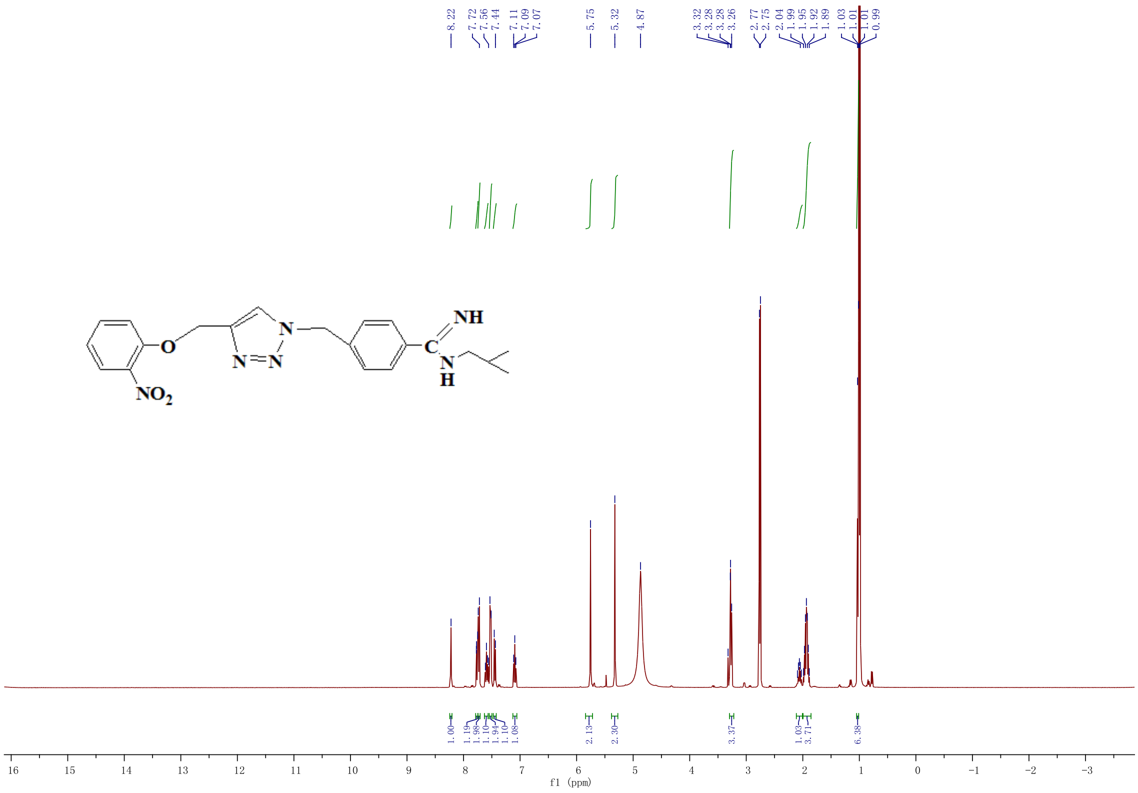

^{1}H-NMR spectrum of compound 14

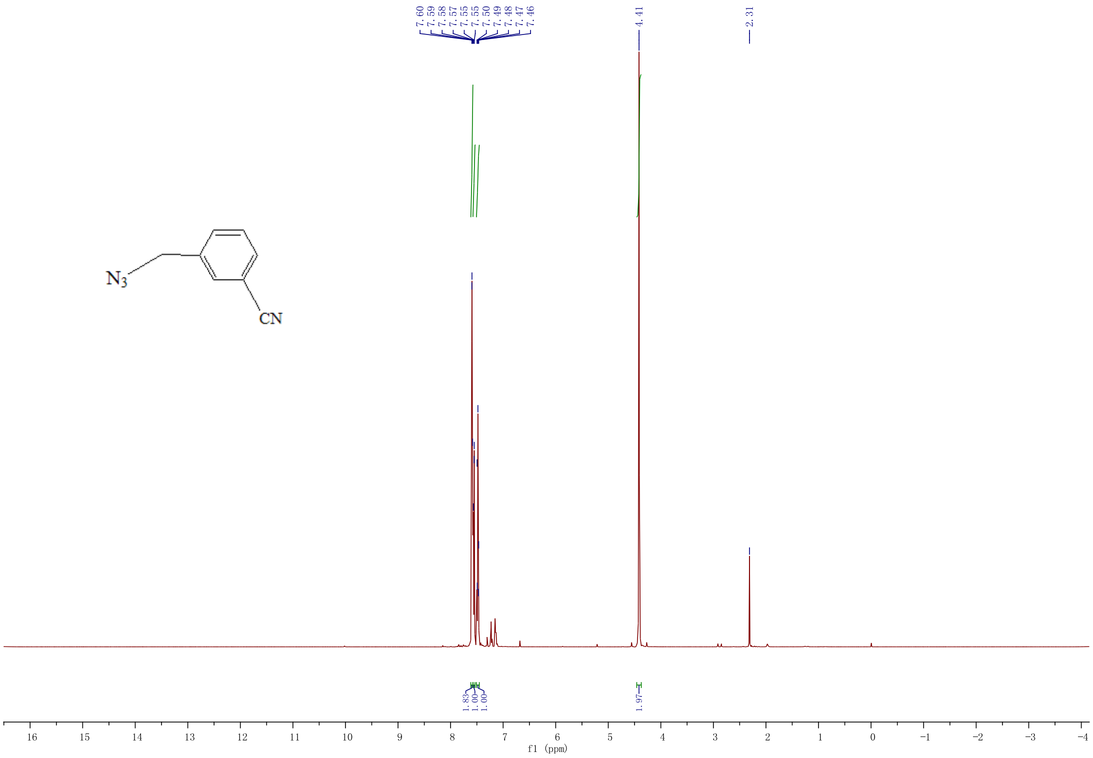

^{13}C-NMR spectrum of compound 14

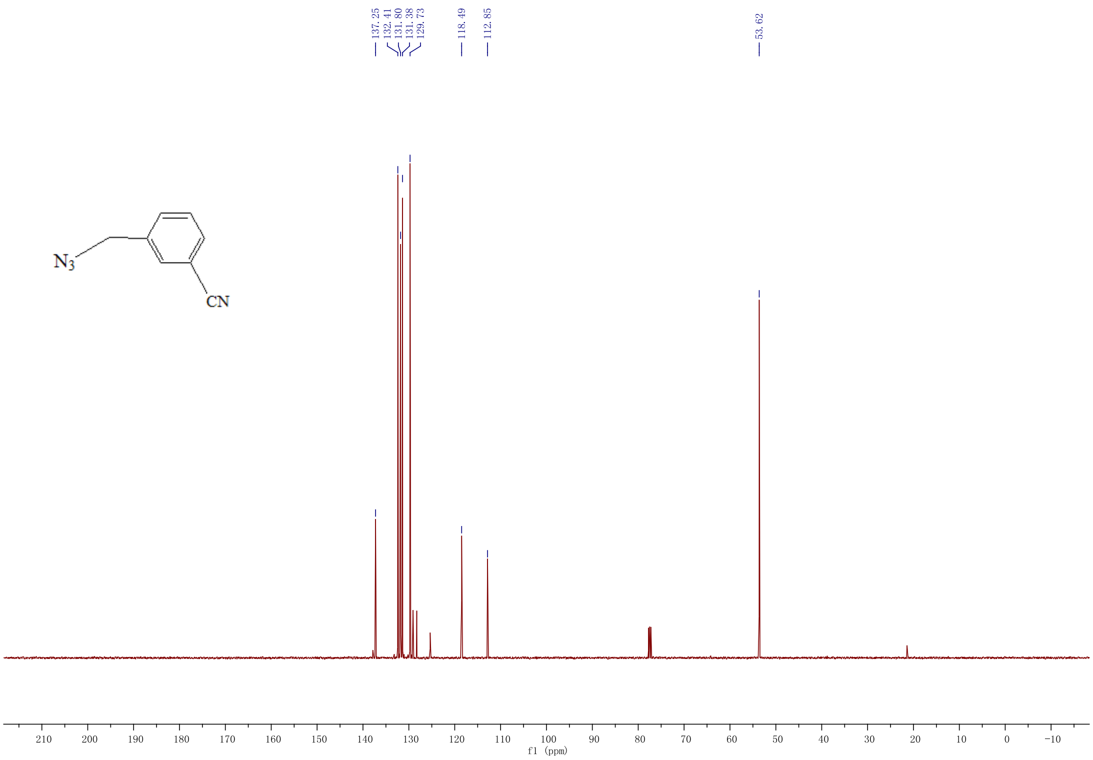

^{1}H-NMR spectrum of compound 15a

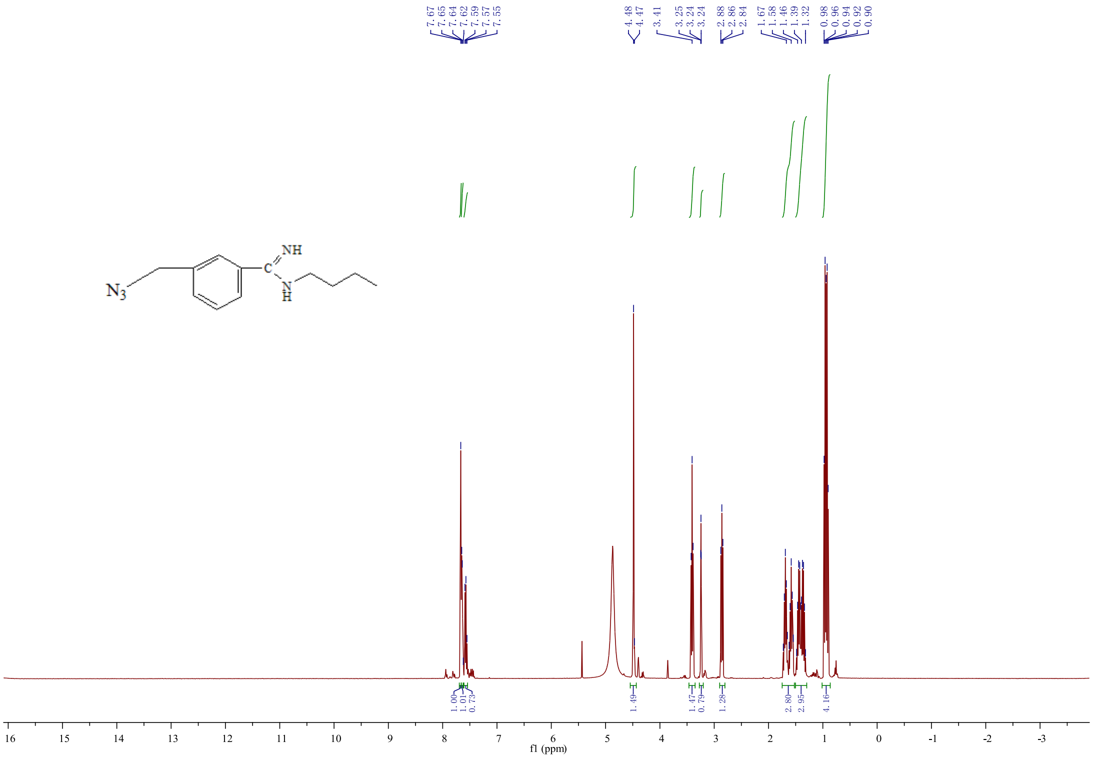

^{13}C-NMR spectrum of compound 15a

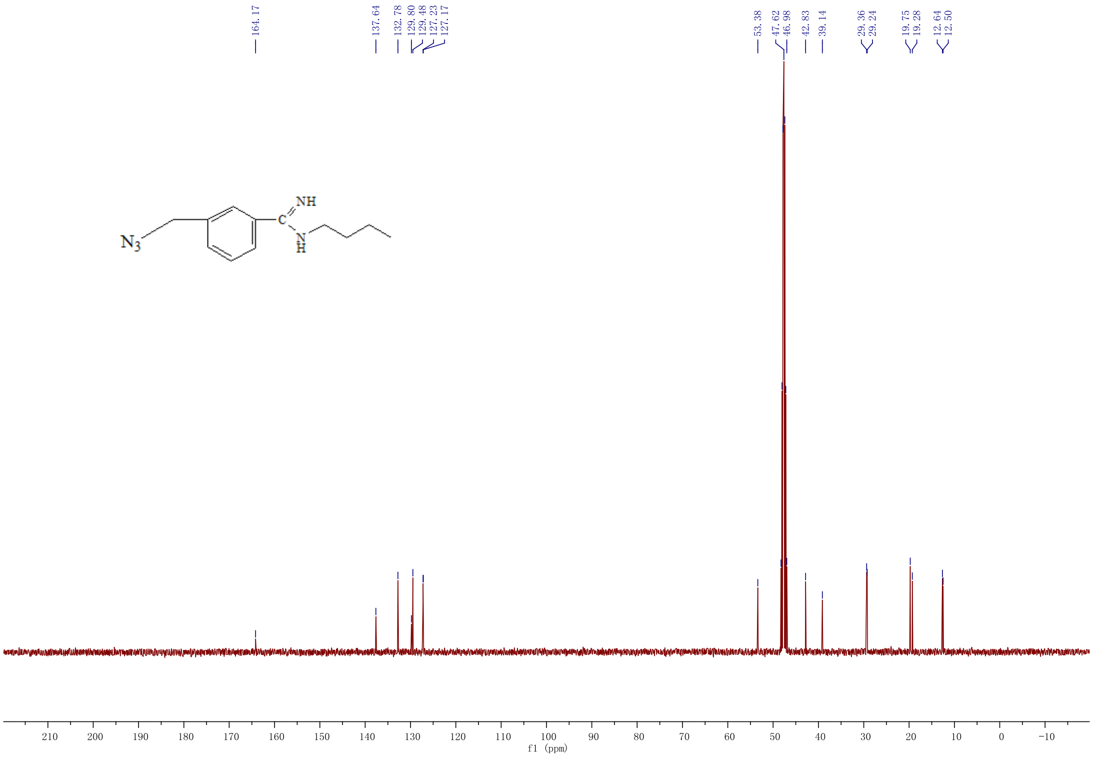

^{1}H-NMR spectrum of compound 15b

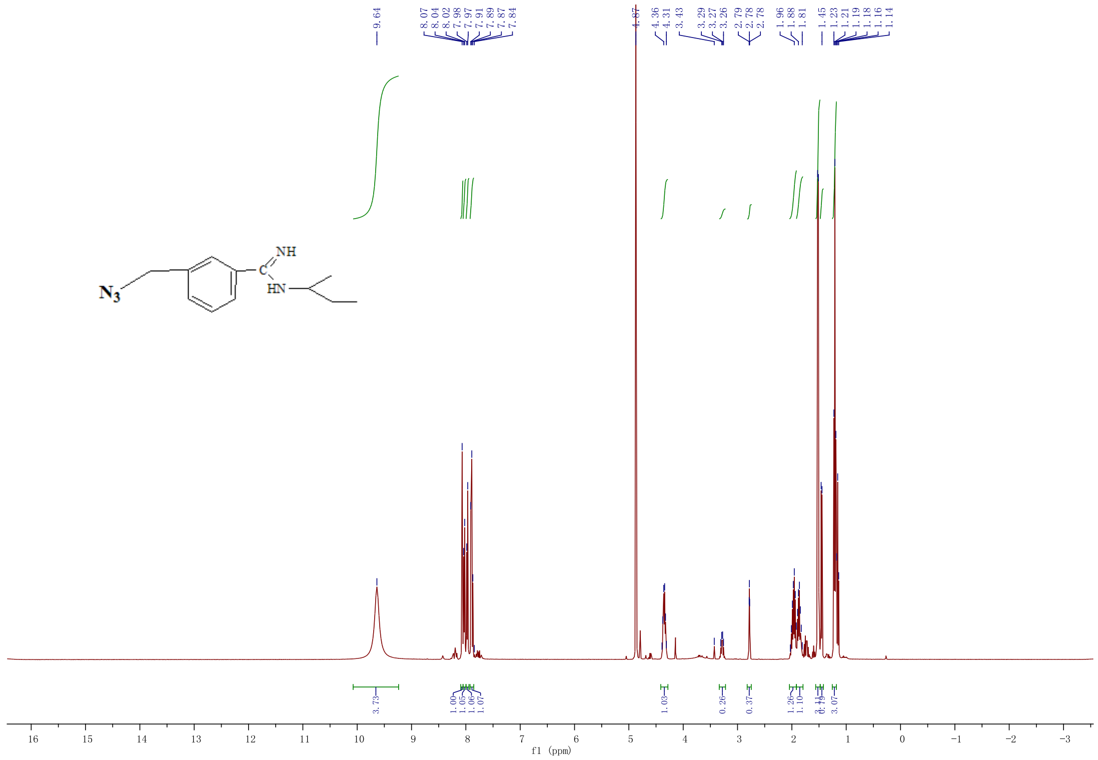

^{13}C-NMR spectrum of compound 15b

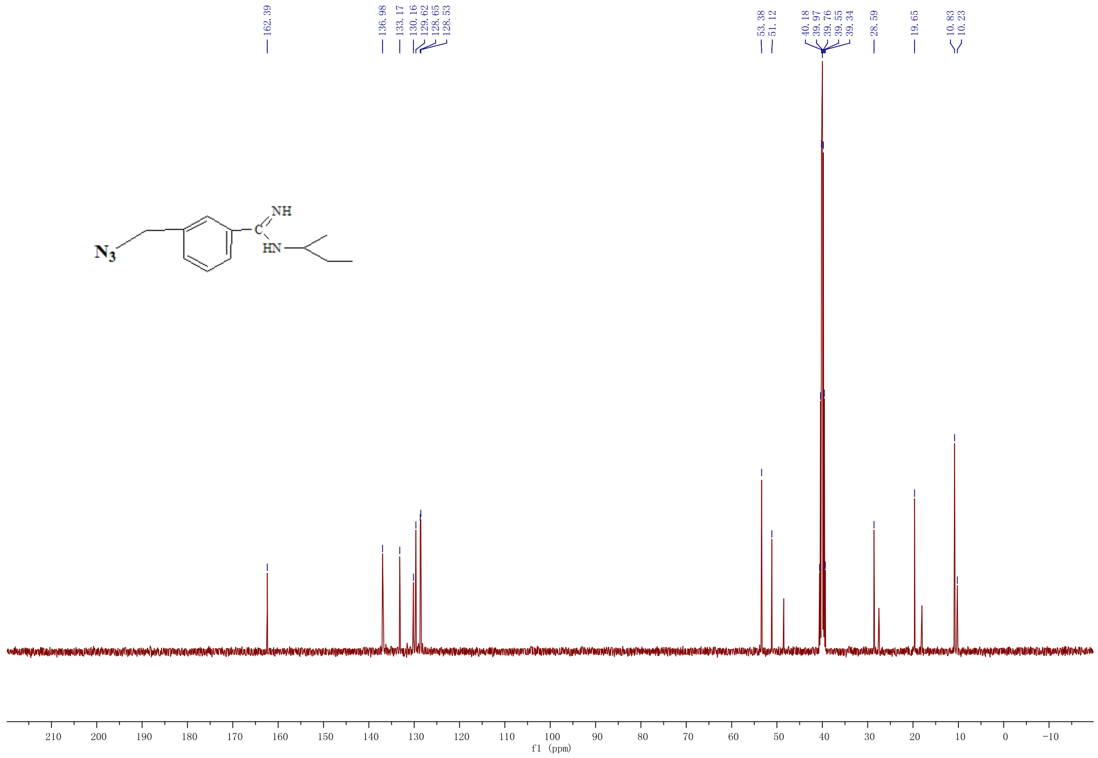

^{1}H-NMR spectrum of compound 16a

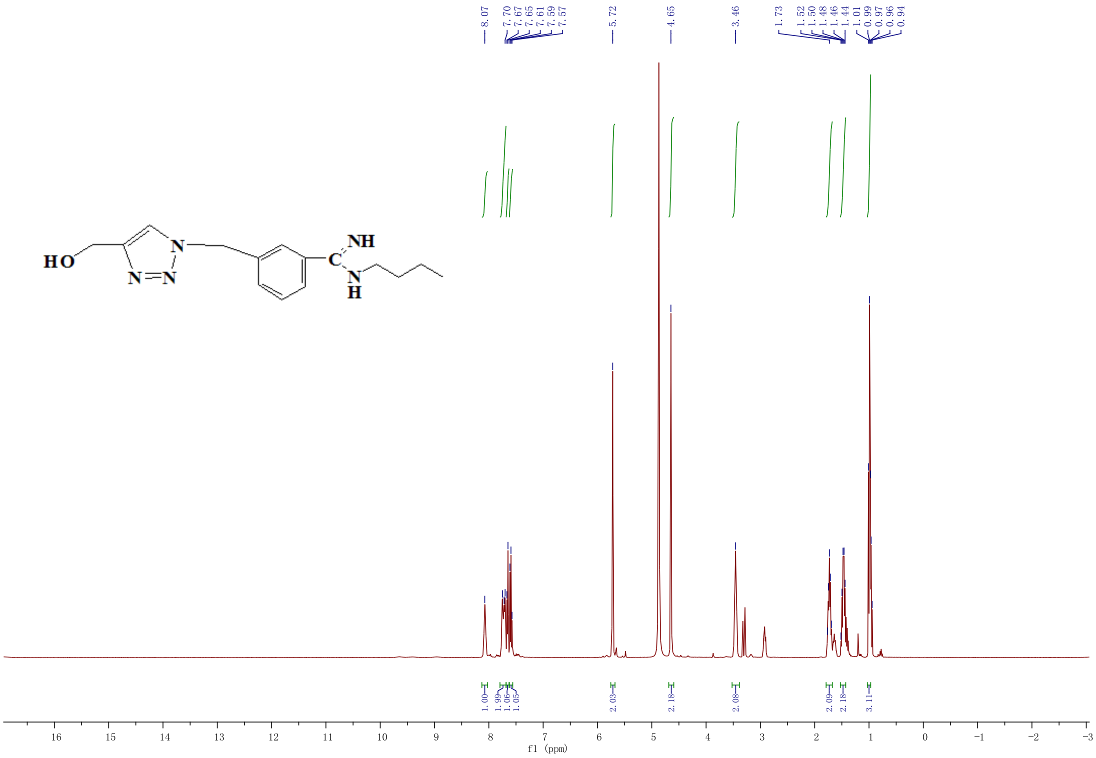

^{13}C-NMR spectrum of compound 16a

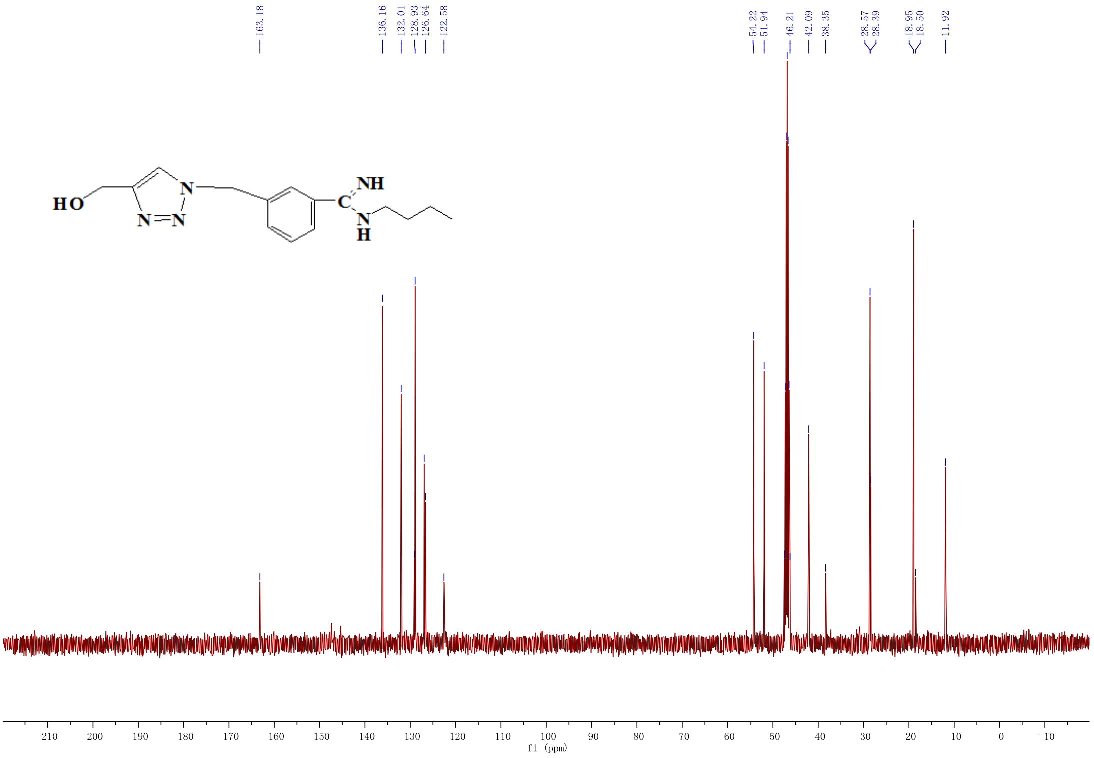

^{1}H-NMR spectrum of compound 16b

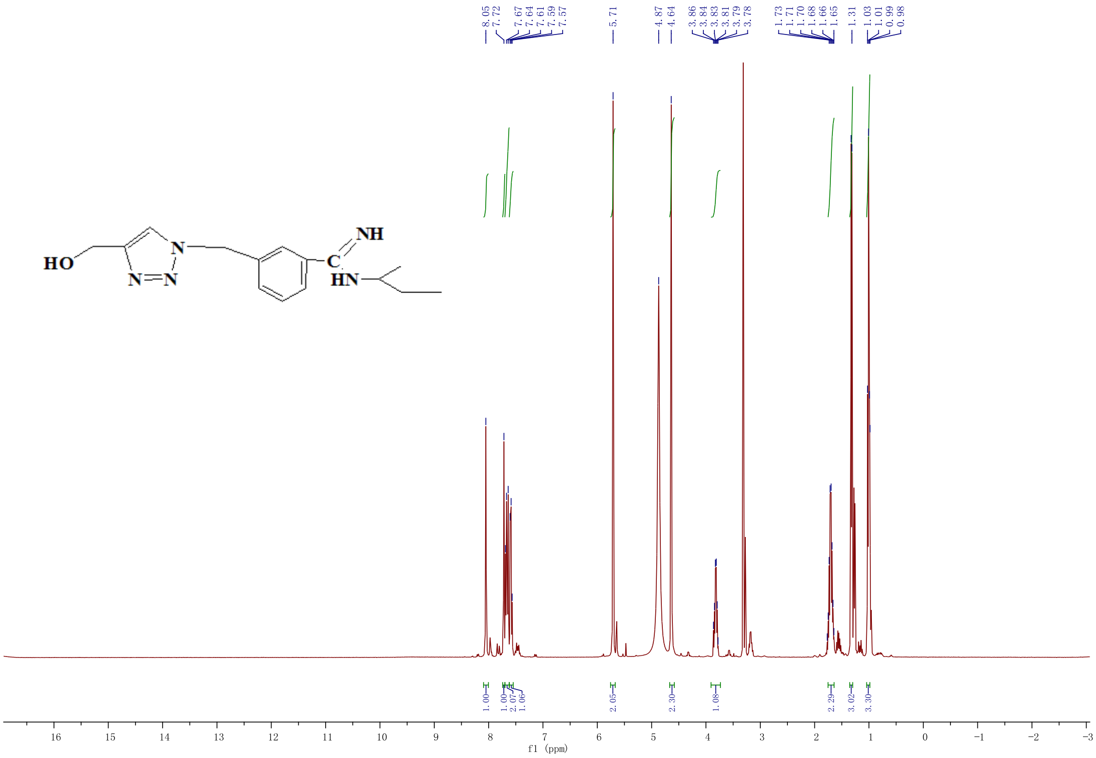

^{13}C-NMR spectrum of compound 16b

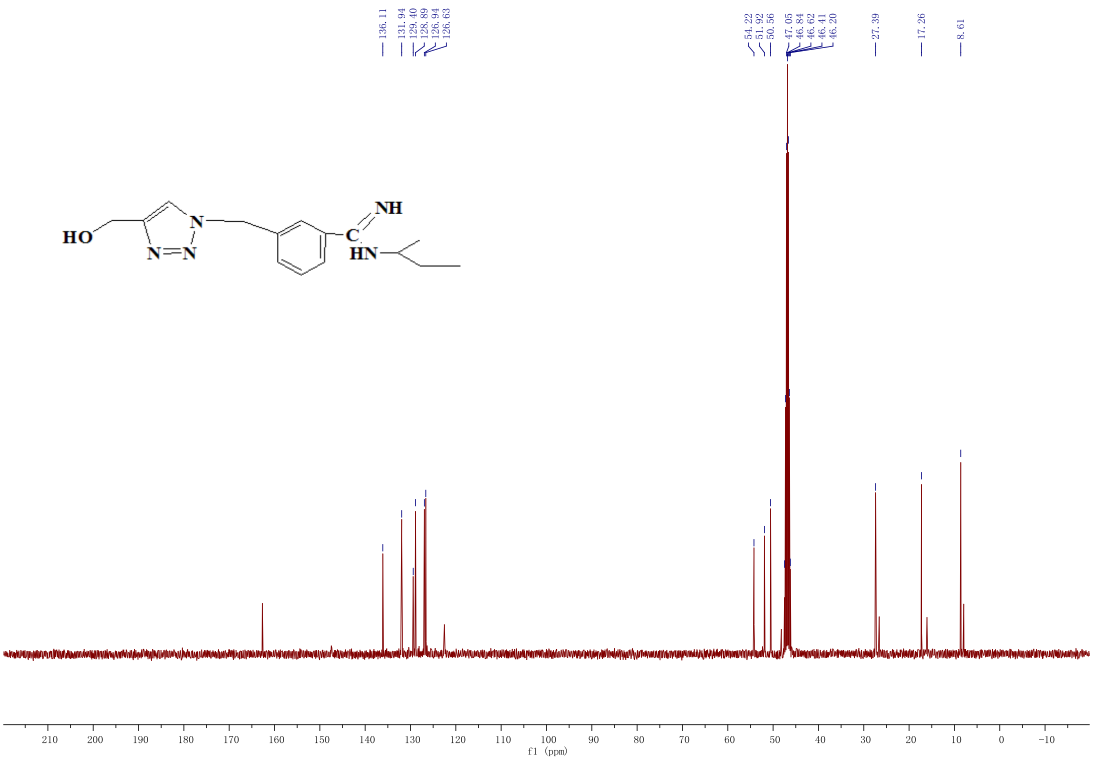

^{1}H-NMR spectrum of compound 17a

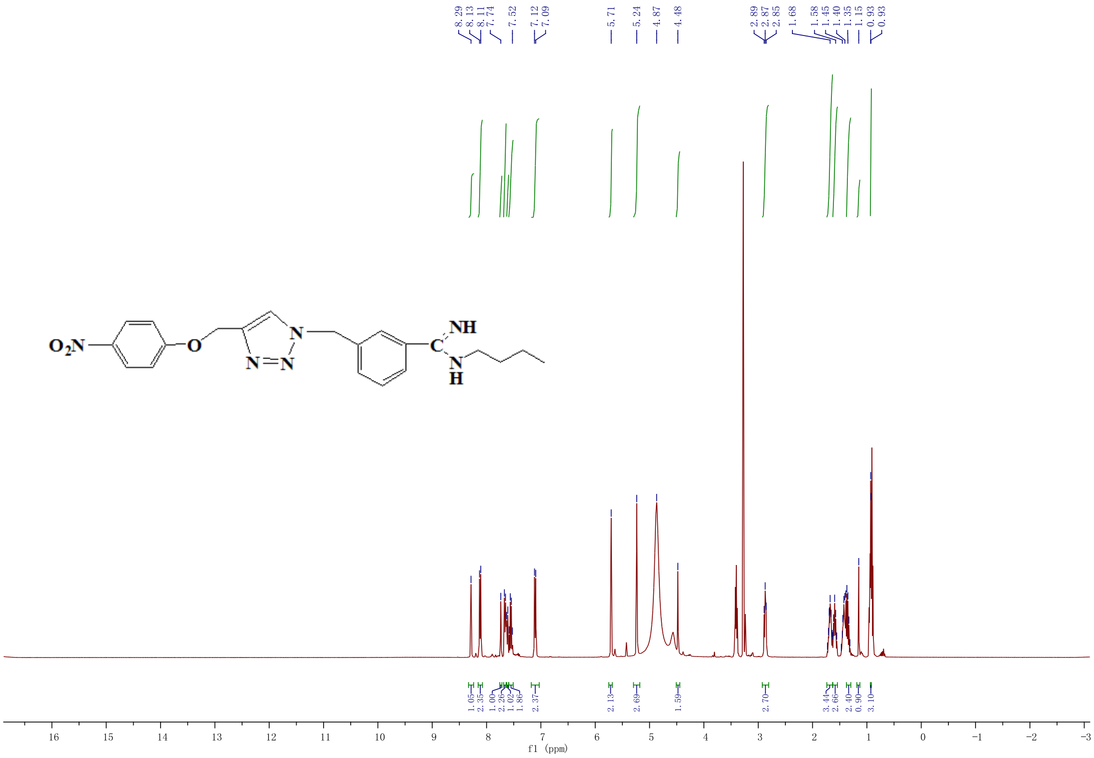

^{13}C-NMR spectrum of compound 17a

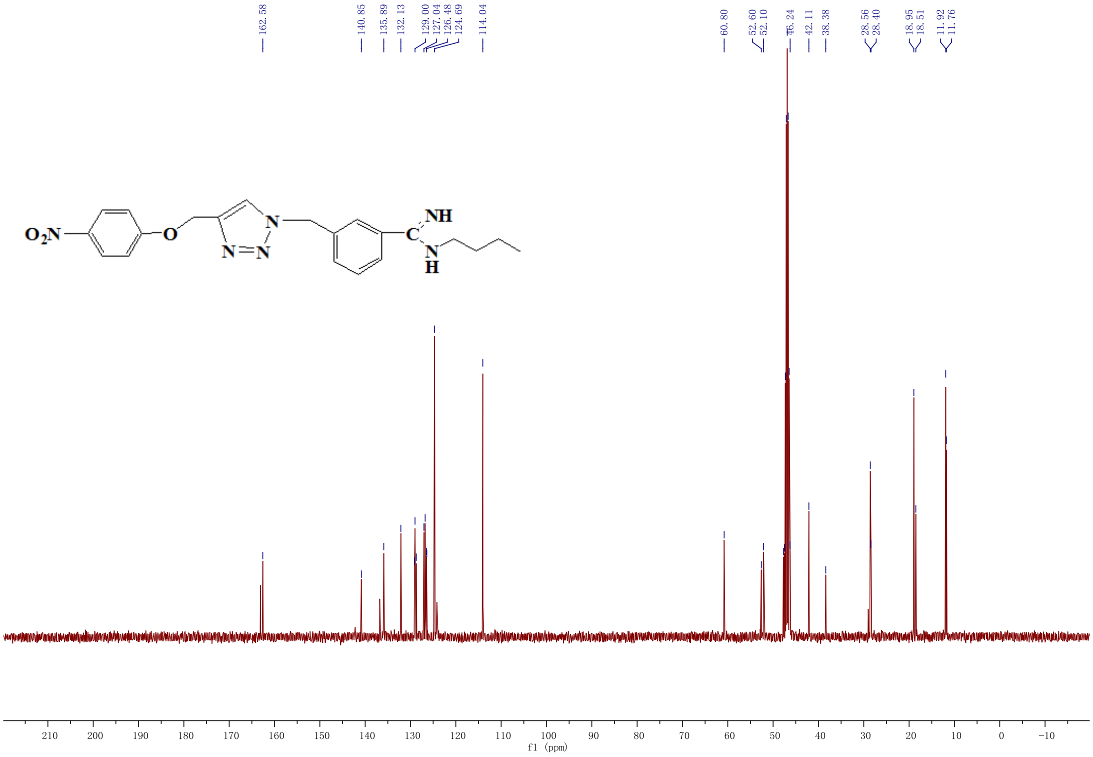

^{1}H-NMR spectrum of compound 17b

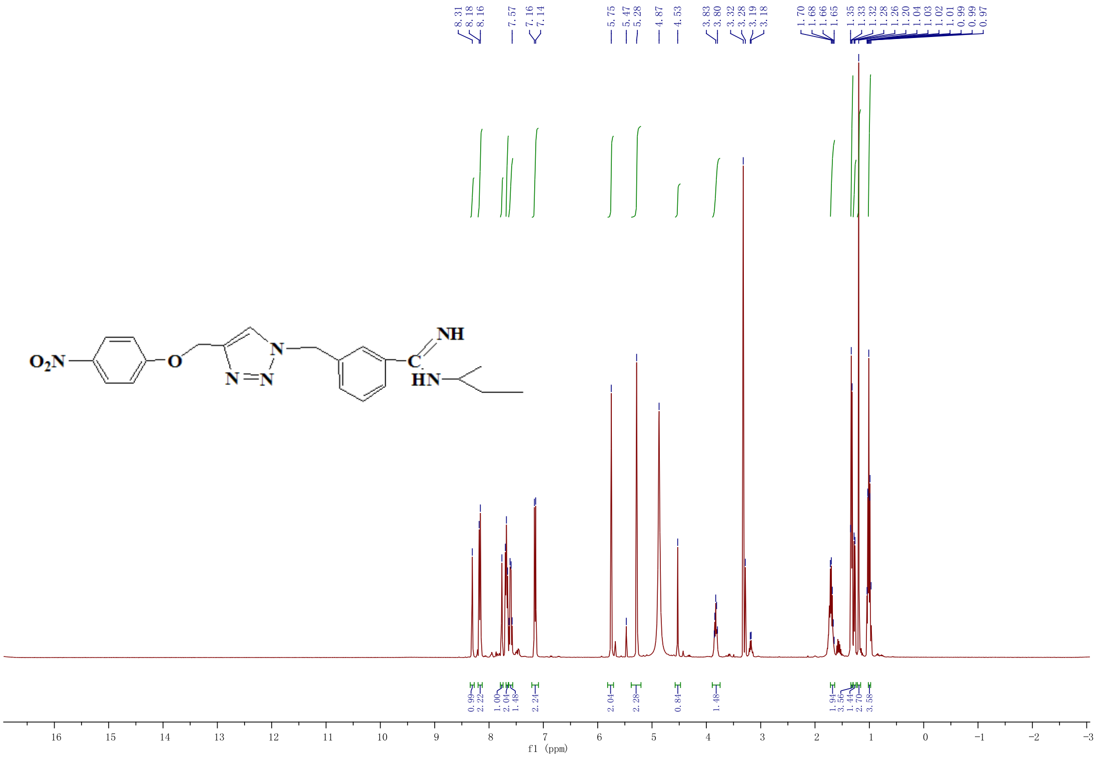

^{13}C-NMR spectrum of compound 17b

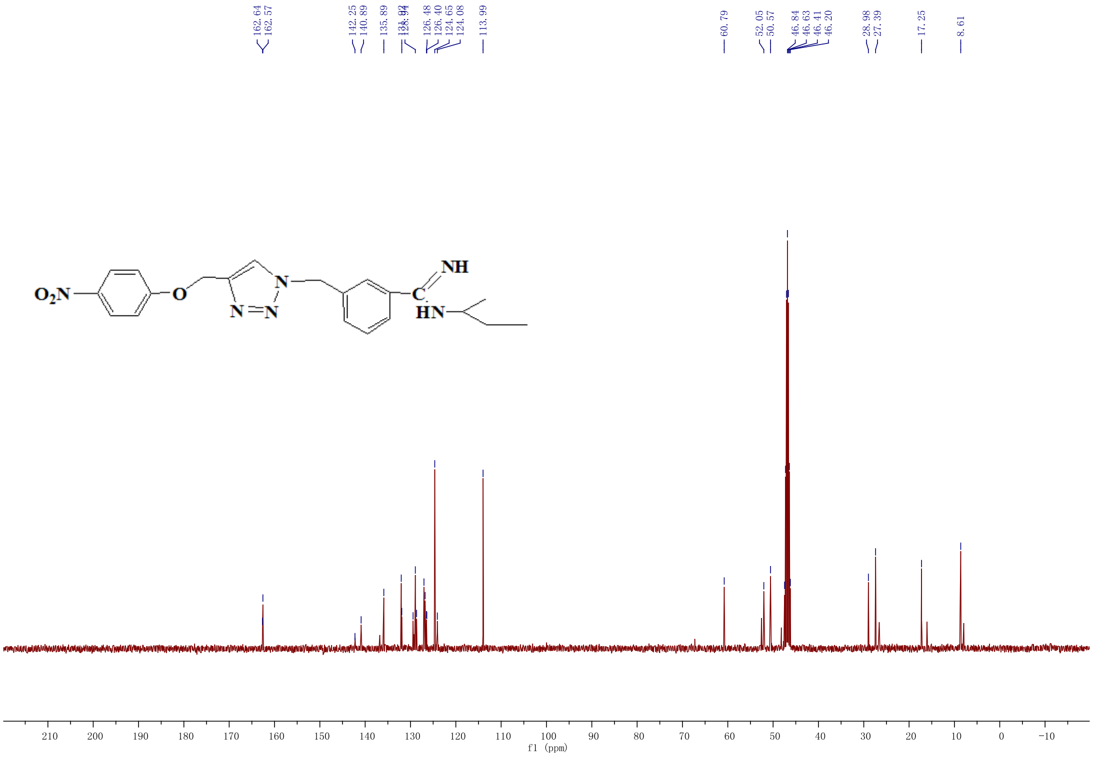

Supplement: Supplementary file 1 [file molecules-19-05674-s001.pdf]
